# Experimental study of local anesthetic and antiarrhythmic activities of fluorinated ethynylpiperidine derivatives

**DOI:** 10.1590/1414-431X2024e13429

**Published:** 2024-07-29

**Authors:** E.M. Satbayeva, S.S. Zhumakova, M.D. Khaiitova, U.S. Kemelbekov, F.M. Tursunkhodzhaeva, A.A. Azamatov, Sh.N. Tursymbek, V.Kh. Sabirov, T.S. Nurgozhin, V.K. Yu, T.M. Seilkhanov

**Affiliations:** 1 Department of Pharmacology, School of General Medicine-1, Asfendiyarov Kazakh National Medical University, Almaty, Republic of Kazakhstan; 2 Laboratory of Synthetic and Natural Medicinal Compounds Chemistry, A.B. Bekturov Institute of Chemical Sciences, Almaty, Republic of Kazakhstan; 3 Department of Pharmacology and Toxicology, S.Yu. Yunusov Institute of the Chemistry of Plant Substances, Academy of Sciences of the Republic of Uzbekistan, Tashkent, Republic of Uzbekistan; 4 Research Laboratory of Medicinal Plants, South Kazakhstan Medical Academy, Shymkent, Republic of Kazakhstan; 5 Laboratory of Structural Chemistry, Tashkent State Technical University, Tashkent, Republic of Uzbekistan; 6 Laboratory of Engineering Profile NMR Spectroscopy, Shokan Ualikhanov Kokshetau University, Kokshetau, Republic of Kazakhstan

**Keywords:** Piperidines, Toxicity, Local anesthetic activity, Antiarrhythmic activity, Molecular docking

## Abstract

The chemical structure of piperidine has a unique ability to combine with other molecular fragments. This fact makes it possible to actively use it as an effective basis for the creation of new drug-like substances. Thus, the aim of the current investigation was to study the acute toxicity, local anesthetic potency, and antiarrhythmic activity of the two new synthesized piperidine derivatives under laboratory codes LAS-286 and LAS-294 (local anesthetic substances). The Bulbring & Wajda animal model and method of determining the nociception threshold during electrical stimulation was used to investigate the action of the substance during infiltration anesthesia. An antiarrhythmic activity was observed by the aconitine-induced rat arrhythmia model. Additionally, these compounds were studied in relation to molecular docking to delineate the structure-activity relationships. The tested piperidine derivatives had a low toxicity in the subcutaneous and intravenous administration routes. The experimental results showed a higher prolonged and pronounced local anesthetic activity for LAS-286 at a 0.5% concentration, compared to the reference preparations. The low dosage of 0.1 mg/kg of LAS-294 demonstrated a pronounced preventive antiarrhythmic effect in 90% of cases on the development of mixed arrhythmia, caused by aconitine. The results of molecular docking confirmed a higher binding affinity of the tested piperidines with the Na_v_1.4 and Na_v_1.5 macromolecules. The results of the present study are very promising, because these piperidines have shown a high biological activity, which can suggest a potential therapeutic application in the future.

## Introduction

Pain is the most common symptom that accompanies the course of various pathological processes and injuries and is expressly present during medical procedures and surgical interventions (1). Thus, a pain syndrome cannot only cause a deterioration in the quality of life and reduce the ability to work, but can also lead to disability, and in some cases, death (2). Nociceptive pain, which occurs in the presence of intense stimuli such as surgery and clinical procedures, must be suppressed with analgesic medications. Local and general anesthetics or high doses of opioids are usually used for this purpose (3). Local anesthetics act on the nerve endings or around nerve trunks, temporarily eliminating local sensitivity (mainly pain) in a state of preserved consciousness. The mechanism of action of this group of preparations is associated with a reversible block in the generation and conduction of nerve impulses (4,5). The use of local anesthetics is widespread in clinical practice ranging from minor injuries and biopsies to long and complex operations. In clinical practice, preference is given to the use of local anesthetics due to their high level of safety, local action, and good tolerability (6).

Taking into account the fact that local anesthetics are considered safe compared with other analgesic drugs, some potential limitations exist for their clinical application. These include allergies, resistance, tachyphylaxis, complications of a toxic, allergic and locally irritating nature, and a limited use in inflamed tissues. The relatively short duration of action of local anesthetics, lasting from a few minutes to several hours, may also limit their use (7). For this reason, the issues of creating new highly effective, long-acting and low-toxic local anesthetics, and their use for effective anesthesia in various branches of medicine, are relevant today. A renewed interest in the development of anesthetic drugs also results from the new demands for a sufficient depth and duration of action in modern clinical practice (8).

The blocking of ion channels, which underlies the mechanism of action of local anesthetics, is directly related to the implementation of an antiarrhythmic activity. An example of this is the local anesthetic lidocaine, which is an effective antiarrhythmic drug, used for ventricular arrhythmia. A study using Rosetta structural computer modeling has revealed that both the antiarrhythmics and local anesthetics share a common receptor site on the cardiac sodium channel (9-11).

New piperidine derivatives are of great interest in this area of research as new local anesthetic and antiarrhythmic agents. Due to their unique chemical structure and ability to bind to various molecular moieties, piperidine derivatives are widely used for the development of new compounds (12). The effectiveness of many substituted piperidine derivatives has been confirmed by numerous studies examining a wide range of pharmacological activities (13,14). The piperidine ring is an important component of the molecular structure of the widely used local anesthetics bupivacaine and ropivacaine (15). The preparations with an analgesic activity, promedol and fentanyl, are also piperidine derivatives (16).

Clinical trials of 1-(2-ethoxyethyl)-4-ethynylpiperidin-4-yl benzoate hydrochloride (kazcaine-experimental substance), jointly developed in the laboratory of the Institute of Chemical Sciences and Pharmacology in the Department of Kazakh National Medical University showed its high effectiveness as both an anesthetic and an antiarrhythmic (17-20). Clinical trials investigating kazcaine are currently in progress. To clarify the effect of introducing fluorine into the kazcaine molecule, we synthesized its *o-* and *m-*fluorine benzoic analogues. Accordingly, the present study aimed to perform *in silico* and *in vivo* studies on experimental animal models to investigate the local anesthetic activity and antiarrhythmic effect of the new substances.

## Material and Methods

### Chemical research and structural investigation of the compounds

The synthesis of new piperidine derivatives under laboratory codes LAS-286 and LAS-294 (local anesthetic substances) was carried out in the Laboratory of Chemistry of Synthetic and Natural Medicinal Compounds of A.B. Bekturov Institute of Chemical Sciences JSC (Almaty). The presence of a fluorine atom in the molecule of organic compounds increases their bioavailability, metabolic stability, lipophilicity, and also improves the ability of these substances to interact with the target proteins (21). As the study objects, *m-* and *o-*fluorobenzoate esters of 1-(2-ethoxyethyl-4-ethynylpiperidin-4-ols) were synthesized, which was previously described in the study of these compounds as potential antimicrobial agents (22). The synthesized fluorobenzoates are fluorine analogues of Kazcaine (1-(2-ethoxyethyl-4-ethynyl-4-benzoyloxypiperidine hydrochloride), which has shown excellent properties as a local anesthetic (19,20). The *m-* and *o-*isomers were obtained by fluorobenzoylation of 1-(2-ethoxyethyl-4-ethynylpiperidin-4-ol with a reagent ratio of 1:1 without a solvent and exposure of the mixture to ultrasound for 10 min with a yield of 81.6 and 87.8%, respectively, while their yield when prepared in dioxane (21) were of 47 and 56%, respectively.

The control of the reactions and the purity of the products were monitored using TLC (thin-layer chromatography) analysis on Al_2_O_3_ plates, visualized by the appearance of substance spots with iodine vapor. The eluent used for TLC was a benzene-dioxane mixture (10:1). An elemental analysis was performed using a Rapid Micro N Cube elemental analyzer (Germany). Infrared (IR) spectra were recorded using a Thermo Scientific Nicolet 5700 FTIR spectrometer (USA) with KBr pellets. The ^1^H and ^13^C NMR (nuclear magnetic resonance) spectra of the samples were recorded on a JNM-ECA 400 (Jeol, Japan) spectrometer operating at frequencies of 399.78 MHz (^1^H) and 100.53 MHz (^13^C) in deuterated dimethyl sulfoxide (DMSO-d_6_).

The structures of 1-(2-ethoxyethyl)-4-ethynyl-4-(m-fluorobenzoyloxy)piperidine (LAS-286 base) and its hydrochloride (LAS-286) were determined by the X-ray diffraction study. The unit cell and intensity parameters were obtained on an Xta LAB Synergy single source diffractometer (Rigaku Oxford Diffraction, Japan) equipped with a Hybrid Pixel Array Detector “HyPix3000” (CuKα, λ=1.54184 Å). The structures were deciphered and refined by the direct method, using the SHELXL 2019/3 software package (23). The positions of hydrogen atoms were calculated geometrically and refined using the rider model with U_iso_=nU_eq_ of the bearing atom (n=1.5 for the methyl groups, n=1.2 for the other hydrogen atoms). The structures were refined, using the F^2^ full-matrix least squares method in the anisotropic approximation for the non-hydrogen atoms. The atom coordinates and other parameters of the LAS-286 base and LAS-286 structures were deposited in the Cambridge Structural Data Bank (http://www.ccdc.cam.ac.uk/data_request/cif) CCDC 2297241 - LAS-286 base (Search Results - Access Structures (cam.ac.uk)) and 2297243 - LAS-286 (Search Results - Access Structures (cam.ac.uk)). The molecular visualization, graphics, and unit cell packaging for LAS-286 base and LAS-286 were performed using the ORTEP-3 (24) and PLATON (25) programs.

### Synthesis of 1-(2-ethoxyethyl)-4-ethynylpiperidin-4-yl 2-fluorobenzoate hydrochloride (LAS-294)

*o-*Fluorobenzoyl chloride (1.75 g, 0.01105 mol) was added to 2.0 g (0.01105 mol) of 1-(2-ethoxyethyl)-4-ethynylpiperidin-4-ol. The mixture was heated and kept at room temperature in an ultrasonic bath for 10 min. Then, water was added to the resulting oily product and treated with potash. The organic part was extracted with benzene, the extract was dried over MgSO_4_, the desiccant was filtered off, and the solvent was evaporated. The product was purified by column chromatography on alumina (eluent: diethyl ether) to obtain 2.74 g (84.8% of the theoretical value) of 1-(2-ethoxyethyl)-4-ethynylpiperidin-4-yl *o-*fluorobenzoate (LAS-294 base), representing a viscous oil. Then, the resulting mass was dissolved in 25 mL of isopropanol and acidified with an isoproponol solution of HCl to pH 3-4. The solvent was evaporated by half, the precipitate was filtered off, washed with diethyl ether, and 1-(2-ethoxyethyl)-4-ethynylpiperidin-4-yl *o-*fluorobenzoate hydrochloride (LAS-294) was obtained in the form of powder, with m.p. 148-150°C. LAS-294 base: Calculated for C_18_H_22_FNO_3_: C, 67.69; H, 6.94; F, 5.95; N, 4.39. Found: C, 67.67; H, 6.99; F, 5.93; N, 4.35. LAS-294 (hydrochloride): Calculated for C_18_H_23_ClFNO_3_: C, 60.76; H, 6.52; Cl, 9.96; F, 5.34; N, 3.94; O, 13.49 Found: C, 60.73; H, 6.58; N, 3.93.

IR spectra of 1-(2-ethoxyethyl)-4-ethynylpiperidin-4-yl o-fluorobenzoate (LAS-294 base) and 1-(2-ethoxyethyl)-4-ethynylpiperidin-4-yl o-fluorobenzoate hydrochloride (LAS-294) are shown in Supplementary Figures S1 and S2.

NMR spectra (^1^Н NMR, ^13^C NMR, COSY, HMQC, HMBC) of 1-(2-ethoxyethyl)-4-ethynylpiperidin-4-yl *o*-fluorobenzoate (LAS-294 base) in DMSO-d_6_ are shown in Supplementary Figures S3-S7.

### Synthesis of 1-(2-ethoxyethyl)-4-ethynylpiperidin-4-yl *m*-fluorobenzoate hydrochloride (LAS-286)

3-Fluorobenzoyl chloride (3.62 g, 0.0228 mol) was added to 1.5 g (0.0076 mol) of 1-(2-ethoxyethyl)-4-ethynylpiperidin-4-ol. The mixture was heated and kept at room temperature in an ultrasonic bath for 10 min. Then, water was added to the resulting mixture, and the mixture was treated with potash. The organic part was extracted with benzene, the extract was dried over MgSO_4_, the desiccant was filtered off, and the solvent was evaporated. The product was purified by column chromatography on alumina (eluent: diethyl ether) to obtain 2.34 g (87.8% of the theoretical value) of 11-(2-ethoxyethyl)-4-ethynylpiperidin-4-yl *m-*fluorobenzoate (LAS-286 base) as white crystals with m.p. 57-58°C. Then, the resulting crystals were dissolved in 25 mL of isopropanol and acidified with a propanol solution of HCl to pH 3-4, the solvent was evaporated by half, the precipitate was filtered off, washed with diethyl ether, and 1-(2-ethoxyethyl)-4-ethynylpiperidin-4-yl *m*-fluorobenzoate hydrochloride (LAS-286) was obtained in the form of powder with m.p. 158-159°C. LAS-286 base: Calculated for C_18_H_23_ClFNO_3_: C, 67.69; H, 6.94; F, 5.95; N, 4.39. Found: C, 67.66; H, 6.96; F, 5.94; N,4.34. LAS-286 (hydrochloride): Calculated for C_18_H_23_ClFNO_3_: C, 60.76; H, 6.52; Cl, 9.96; F, 5.34; N, 3.94; O, 13.49. Found: C 60,74; Н 6,57; N, 3.93.

IR spectra of 1-(2-ethoxyethyl)-4-ethynylpiperidin-4-yl *m-*fluorobenzoate (LAS-286 base) and 1-(2-ethoxyethyl)-4-ethynylpiperidin-4-yl *m-*fluorobenzoate hydrochloride (LAS-286) are shown in Supplementary Figures S8 and S9.

NMR spectra (^1^H NMR, ^13^C NMR, COSY, HMQC, HMBC) of 1-(2-ethoxyethyl)-4-ethynylpiperidin-4-yl *m-*fluorobenzoate (LAS-286 base) in DMSO-d_6_ are shown in Supplementary Figures S10-S14.

### *In silico* study

In order to determine the preclinical research directions, the chemical structures of the new piperidine derivatives were analyzed using computer programs. The identification of the probable targets was carried out using the online web tool SwissTargetPrediction on the SwissDrugDesign software platform, (http://www.swisstaretprediction.ch/). Entering the chemical formula of the studied substances made it possible to predict the most likely targets of the protein structure. The prediction was made on the basis of a search for similarity of ligands and descriptors with similar molecules, which were entered into the program (26).

The expected range of the biological activity of the piperidine derivatives was determined using the online PASS program on the Way2Drug web resource platform (http://way2drug.com/). The operating principle of this program is to process the entered chemical structure and analyze it using a prediction algorithm based on the Bayesian approach. An assessment of the potential presence of the effect was made according to the probability of the presence (P_a_) and absence (P_i_) indicators, calculated by the program. The index value of 0.3<P_a_<0.7 indicates the highest probability of detecting this type of activity at the experimental stages of the research (27).

Based on the study results, molecular docking was carried out to analyze the interaction of the molecules of the studied compounds with the macromolecules of the potential targets-voltage-dependent sodium channels. The structures of the studied compounds were generated using Marvin JSpowered by ChemAxon and saved in the MOL2 format (Tripos Mol2). These were then converted to PDBQT files using PyRx. The sodium channel macromolecules were downloaded from the Protein Data Bank in Europe and the Protein Data Bank (USA, https://www.rcsb.org/), and also saved in the PDBQT format. For docking with Na_v_1.4, we used a grid with x, y, and z dimensions of 25.0, 25.0, and 65.9 Å, respectively. For Na_v_1.5, we used the grid sizes of 25.0, 25.0, and 72.3 Å in the x, y, and z dimensions, respectively. Docking was performed using Autodock Vina with an exhaustiveness parameter of 8. After calculating the molecular docking results, we selected the positions in which the compounds formed the strongest bonds and that also presented root mean square deviation (RMSD) values equal to 0.

### Experimental animals

The experimental studies on acute toxicity were carried out on outbred laboratory mice (8-9-week-old, weighing 20-25 g) and rats (7-8-week-old, weighing 185-250 g) of both sexes. The experiments to study local anesthetic activity during the infiltration anesthesia were carried out on male guinea pigs (mature animals, weighing 350-400 g) and outbred male rabbits (mature animals, weighing 2500-3000 g). The research was carried out in the Life Science laboratory of Asfendiyarov Kazakh National Medical University (KazNMU). The laboratory animals were provided by the KazNMU vivarium.

The study of antiarrhythmic activity was carried out in the laboratory of the Department of Pharmacology and Toxicology of S.Yu. Yunussov Institute of Chemistry of Plant Substances of the Academy of Sciences of the Republic of Uzbekistan in Tashkent on outbred laboratory male rats (8-10 weeks old, weighing 180-300 g), obtained from the vivarium of this institute.

All laboratory animals were quarantined for 2 weeks and adapted during this period to the conditions of the upcoming experiments. The animals (each sex differently) were kept in specialized cages in compliance with the necessary hygienic conditions at a temperature of 25±2°C and with good air circulation in a natural 12-h day-night light regime with constant free access to clean water and standardized food for each type of animal. Appropriate marking for the subsequent identification of the animals was carried out using a permanent marker.

### Ethics approval

All research with the laboratory animals was carried out in accordance with the Order of the Minister of Health of the Republic of Kazakhstan No. KRDSM-255/2020 “On approval of the rules for conducting preclinical (non-clinical) studies and requirements for the preclinical bases for assessing the biological effect of medical devices” (December 11, 2020), and the rules of the European Convention for the Protection of Vertebrate Animals and Directive 2010/63/EU. The Protocol for this study was approved and monitored by the Ethics Committee of Asfendiyarov Kazakh National Medical University (Protocol No. 14 (120), dated 10.28.2021, with permission to extend the study - Protocol No. 1 (137), dated 01.31.2023).

### Acute toxicity study

The experiments to study acute toxicity (28) were carried out on healthy male and female outbred laboratory mice and rats randomly divided into experimental and control groups of 6 laboratory animals (3 females and 3 males) in each group. The aqueous solutions of the test compounds and reference drugs in increasing concentrations were administered once. Sterile water for injection (Novosibkhimpharm Co., Russia) was used as a solvent. The solutions of the compounds were injected subcutaneously into the lateral surface of the body of the mice and into the lateral tail vein of the rats. After the administration of the test solutions, all laboratory animals were observed for 14 days to record systemic toxic manifestations in the form of changes in behavioral reactions, motor activity, and metabolism, and the presence of pathological manifestations in the organs and systems. Based on the study results, the average (and standard error) lethal dose (LD_50_) was calculated.

### Local anesthetic activity study

The primary study of local anesthetic activity during the infiltration anesthesia was carried out using the Bulbring and Wajda model in male guinea pigs (29,30). Each experimental group included 6 randomly separated laboratory animals. The day before the test, the hair in the back area was first removed. The 0.5% aqueous solutions were administered intradermally (wheal method) in a volume of 0.25 mL at 4 points at the corners of a square with a side of 3 cm (Supplementary Figure S15).

The test compound was injected into the anterior and posterior points, and a reference solution was injected into the remaining parallel points. The injected area was marked with ink. The presence or absence of sensitivity at the injection site was assessed every 5 min by touching with the injection needle in series of 6 touches at intervals of 3-4 s. Based on the results of the experiments in each series, the total number of needle touches that did not cause a reaction in the animal (skin twitching) for 30 min (anesthesia index), the duration of full anesthesia, and the total duration of the anesthetic effect were recorded.

An in-depth study of local anesthetic activity during the infiltration anesthesia of the abdominal wall in rabbits was carried out by determining the nociception threshold during electrical stimulation. The experiments were performed on non-anesthetized male rabbits, with 6 animals in each group (28,30). The animal was first fixed by its paws in a supine position. Before the start of the experiment, the skin of the abdomen was freed from hair at the level of the middle third of the lateral surfaces. Electrical stimulator (Medistim Co., Russia) electrodes soaked in cotton balls with a solution of 0.9% NaCl were fixed on the shaved areas. Next, the pain sensitivity threshold was determined by applying minimal stimulation with electric current pulses (duration 0.3 ms, frequency 50 Hz, amplitude 5-25 V) accompanied by the appearance of a response, recorded in the form of a change in the rhythm and amplitude of the animal’s breathing using a veterinary multiparameter monitor OLV-VM12 (Zhengzhou Olive Electronic Technology Co., Ltd., China). At the location of the electrodes, the 0.5% solution of the test substance was injected intradermally in the volume of 0.5 mL and subcutaneously in the volume of 2 mL. Next, the nociceptive reaction was tested in response to the stimulation after 3, 5, 10 min, etc. after the administration of the substance (Supplementary Figure S16).

The time of development of anesthesia and its depth (in percentage) and duration were assessed by changing the threshold of the reaction upon the electrical stimulation of the skin area, infiltrated with the solution of the test substance. The effect of the substance, which eliminates the reaction to the threshold stimulation, was taken as 20% anesthesia. An increase in the threshold value of irritation by 5 V was considered to be 40% anesthesia, by 10 V - by 60%, by 20 V - by 100%.

Sterile water for injection was used as a solvent for the piperidine derivative substances. The most widely used local anesthetic preparations for infiltration anesthesia with varying severity and duration of effects were selected as reference preparations: procaine (KHIMFARM JSC, Kazakhstan), lidocaine (Borisov factory of medical preparations JSC, Belarus), trimecaine (Zhayik-AS LLC, Kazakhstan).

### Antiarrhythmic activity study

The antiarrhythmic activity was studied using the aconitine model of arrhythmia (28,31). The experiments were carried out on anesthetized (ethaminal sodium at the dose of 50 mg/kg, intraperitoneally) outbred male rats, randomly divided into groups of 10 animals each to study one compound. Previously, the animals have had an ECG recorded in standard lead II, using an electrocardiograph (Schillervet AT-1, Switzerland). To induce an arrhythmia, aconitine was administered into the lateral tail vein at the dose of 12 µg/kg (obtained from Academician S.Yu. Yunussov Institute of Chemistry of Plant Substances of the Academy of Sciences of the Republic of Uzbekistan) (Supplementary Figure S17). To determine the preventive effect of the test compound and reference preparations, the solutions of these substances were administered intravenously in a wide range of doses 5 min before the administration of the aconitine solution. The ECG registration was carried out for 30 s and 1, 2, 3, 4, and 5 min and subsequently every 5 min after the administration of aconitine. In each experimental group, the number of animals with and without arrhythmia and the antiarrhythmic effect were recorded. The antiarrhythmic activity was assessed by the ability to prevent the development of cardiac arrhythmias caused by the administration of aconitine, with the determination of the main parameters of the median effective dose (ED_50_) and the antiarrhythmic index (LD_50_/ED_50_).

Antiarrhythmics of Class I, which have a similar activity mechanism as the compounds under study, were used as reference drugs: procainamide (Organika JSC, Russia) and allapinin (S.Yu. Yunussov Institute of Chemistry of Plant Substances of the Academy of Sciences of the Republic of Uzbekistan).

### Statistical analysis

The data obtained during the study are reported as means±SE or SD. LD_50_ was calculated using the “QuestGraph™ LD50Calculator” (AATBioquest, Inc., Feb. 19, 2023, https://www.aatbio.com/tools/ld50-calculator/), and its standard error was determined (32). The “QuestGraph™ ED_50_Calculator” (AATBioquest, Inc., Feb. 20, 2023 https://www.aatbio.com/tools/ed50-calculator/) was used to determine ED_50_. The statistical significance of differences in the experimental groups were determined using *t*-test when comparing the two groups and one-way analysis of variance (ANOVA) when evaluating multiple groups. SPSS/27.0 software (IBM, USA) for Windows was used for the statistical data processing. A value of P<0.05 was considered statistically significant.

## Results and Discussion

### Results of the synthesis of the new compounds

The target molecules (LAS-286 and LAS-294) were obtained by acylation of 1-(2-ethoxyethyl)-4-ethynyl-4-hydroxypiperidine, with *o-* or *m-* fluorobenzoyl chloride, respectively, by keeping the reaction mixture for 10 min in an ultrasonic bath. The process of obtaining the investigated compounds can be viewed in the Supplementary Figure S18. The chemical structures of the tested compounds are shown in Supplementary Figure S19.

The structures of 1-(2-ethoxyethyl)-4-ethynylpiperidin-4-yl fluorobenzoates (LAS-286 base and LAS-294 base) and their hydrochlorides (LAS-286 and LAS-294) were established by the IR (Supplementary Figures S1, S2, S8, S9) and NMR (Supplementary Figures S3-S7, S10-S14) spectroscopy.

The ^1^H and ^13^C NMR spectra correspond to the structure of the target compounds (LAS-286 base and LAS-294 base). Their structure was also confirmed by two-dimensional NMR spectroscopy COSY (1H-1H), HMQC (1H-13C), and HMBC (1H-13C), which made it possible to establish spin-spin interactions of homo- and heteronuclear nature. The observed COSY (1H-1H) and HMQC (1H-13C) NMR correlations in the molecule are presented in Supplementary Figure S20.

In the IR spectra of the studied fluorine derivatives of piperidine, both in the form of bases and hydrochlorides, there was a strong absorption band of ester carbonyl (C=O) at 1730.9-1717.8 cm^-1^. The triple C≡C bond absorbs infrared light in the region of 2113.2-2105.7 cm^-1^, and the bands at 3301.2-3249.8 cm^-1^ were attributed to the C-H bond at the triple bond.

The structure of 1-(2-ethoxyethyl)-4-ethynyl-4-(m-fluorobenzoyloxy)piperidine (LAS-286 base) and its hydrochloride (LAS-286), established by the X-ray diffraction study are presented in Supplementary Figures S21-S24 and Supplementary Table S1.

LAS-286 base is a molecular fluorine derivative of piperidine - 1-(2-ethoxyethyl)-4-ethynyl-4-(m-fluorobenzoyloxy)piperidine. The structure of LAS-286 base is similar to that of the kazcaine base (17). Many structural data are consistent with each other, especially the conformation of the piperidine ring - “chair”, the bond lengths of the ethoxyethyl radical, the acetylene part, and the position of the m-fluorobenzoyloxyl radical are similar. An important difference is the presence of a fluorine atom F(1), which is in the meta position relative to the acyl fragment, while the bond length with the aromatic ring is C(17)-F(1) 1.322(4) Å, (Supplementary Figure S21). The crystalline structure of the LAS-286 base is shown in Supplementary Figure S22.

LAS-286 is a salt - 1-(2-ethoxyethyl)-4-ethynyl-4-(m-fluorobenzoyloxy) piperidine hydrochloride. The structure of LAS-286 is also similar to the previously described kazcaine hydrochloride (33). The fluorine atom F(1) is in the *meta* position relative to the acyl fragment, and the length of the C(17)-F(1) bond is 1.347(5) Å (Supplementary Figure S23). The molecules in LAS-286 crystal are stabilized due to the similar intramolecular bonds (30). The crystalline structure of LAS-286 is shown in Supplementary Figure S24.

### *In silico* prediction

**Figure 1:**
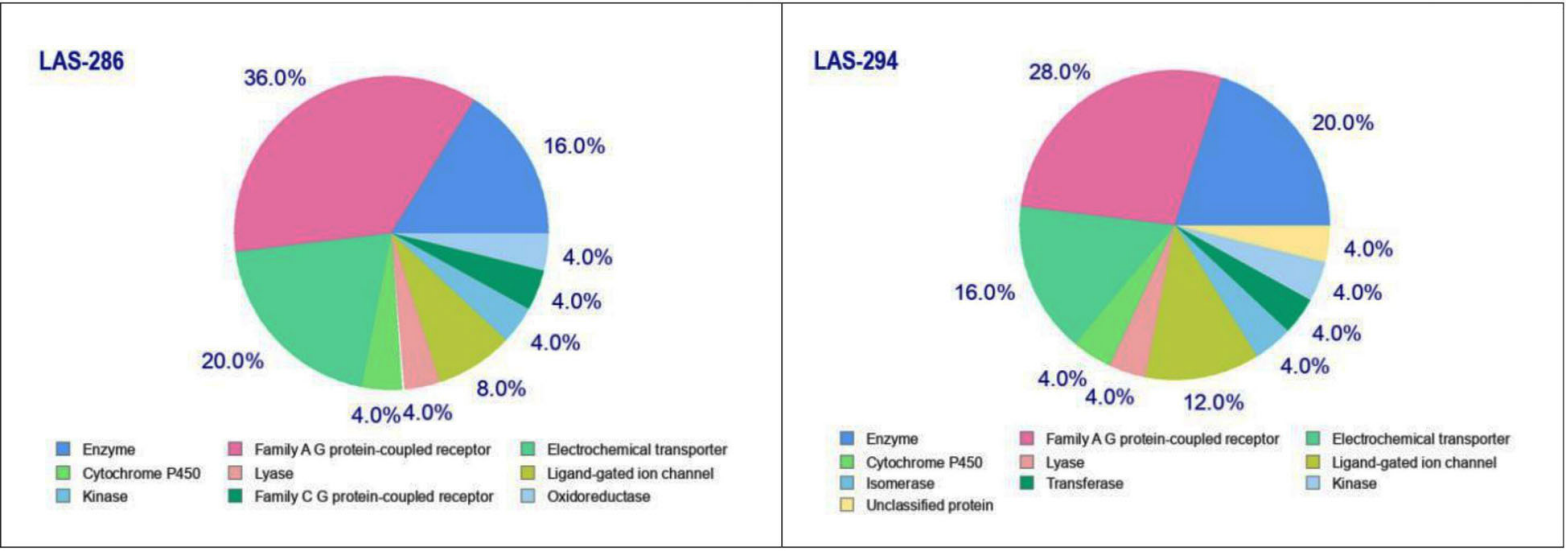
Target classes of LAS-286 and LAS-294 in preclinical research, identified by the SwissTargetPrediction webtool (http://www.swisstargetprediction.ch/).

Taking into account the purpose of our study, it was important to determine the likely targets through which the local anesthetic and antiarrhythmic effects are realized, as well as the presence of a predictor of these effects in the biological activity spectrum. Along with the classical mechanism of action of the local anesthetics on the voltage-gated sodium channels (and to a lesser extent potassium and calcium), the drugs of this group are also able to interact with the ligand-gated channels and G protein-coupled receptors, thereby also causing a local anesthetic activity (7). The SwissTargetPrediction web tool identified the potential targets for the chemical structures of the newly synthesized piperidine derivatives. The results, shown in Figure 1, revealed that the compounds may affect various classes of the targets, including enzymes, cytochrome P450, receptors, transporters, and ion channels.

In general, because of the similar chemical structure, LAS-286 and LAS-294 have probable targets of similar classes, where a more pronounced effect is noted on the family A G protein-coupled receptor (36 and 28%) and electrochemical transporters (20 and 16%). It was important to identify the ligand-gated ion channel class (8 and 12%) with a sufficiently high level of impact for the two studied compounds. A more detailed analysis allowed us to identify the most important targets, most likely associated with pain mechanisms and cardiac arrhythmias. Thus, the delta- and mu-opioid receptors, voltage-gated L-type calcium channel (alpha-1C subunit), in the class of targets ligand-gated ion channel-neuronal acetylcholine receptors, glutamate NMDA receptors, and ATP-sensitive inward rectifier potassium channel were identified as probable targets among the A G family protein-coupled receptors. In addition, the predictive analysis showed the likelihood of LAS-286 influencing the sodium channel protein type II alpha subunit.

When studying the spectrum of biological activity using the PASS program, the derivatives LAS-286 and LAS-294 revealed a high probability of an anesthetic effect (P_a_ 0.524 and 0.422, respectively) and an almost equal probability of a local anesthetic effect (P_a_ 0.377 and 0.327, respectively). In terms of the effect on the cardiovascular system, the analysis showed a significant probability of an antianginal effect for LAS-294 (P_a_=0.528), inferior to LAS-286 (P_a_=0.45).

### Acute toxicity evaluation

The compounds and reference drugs were administered in 3-4% solutions, depending on the dose, in a volume of no more than 1.0 mL. The toxicity indicators based on the results of a series of experiments with the subcutaneous administration on laboratory mice are presented in Table 1.

**Table 1:** Indicators of lethal doses in the acute toxicity study of the piperidine derivatives via the subcutaneous administration route.

Compound/reference drug	LD_16_ (mg/kg)	LD_50_ (mg/kg)	LD_84_ (mg/kg)
LAS-286	1321.9	1447.5±72.3*	1574.7
LAS-294	856.5	1121.1±157.4*	1407.6
Lidocaine	-	230±35.7	-
Procaine	-	480±1.0	-
Trimekaine	-	375±3.1	-

Data reported as means±SE (n=6). *P<0.001 compared to reference drugs (*t*-test).

An analysis of the experimental results has shown that LAS-286 is the least toxic compound, and the LD_50_ values are statistically significant (P<0.001) and much higher than those of procaine by 3 times, trimecaine by 3.8 times, and lidocaine by 6 times. The relative toxicity of LAS-286 varied within 0.16-0.33 of the toxicity of the reference drugs when administered subcutaneously. LAS-294 has also been found to have significantly less toxicity than the reference drugs. The obtained LD_50_ value of the studied compound was statistically significant (P<0.001), more than 5 times higher than that of lidocaine when administered subcutaneously, and more than 2 times higher than those of procaine and trimecaine. The relative toxicity of LAS-294 to that of the reference reparations ranged from 0.19 to 0.40 when administered subcutaneously. The two piperidine derivatives can be classified as toxicity Class 4 according to the OECD (Organisation for Economic Co-operation and Development) classification (34).

The clinical manifestations of poisoning upon the administration of toxic doses of the two compounds were generally similar and differed in the rapid increase in symptoms of intoxication with increasing doses. However, the intoxication phenomena were more pronounced and with a faster rate of increase in LAS-294. With the subcutaneous administration of lower dosages, the changes in the respiratory system, the motor activity, and the sensory perception were observed with a gradual restoration of the physiological functions of the laboratory animals. The increase of the administered dosage led to the appearance of rapidly increasing symptoms of intoxication, the occurrence of convulsive twitching, followed by the death of a number of the animals in the first 30 min after the administration. In the surviving animals, a decrease in the severity of intoxication symptoms was noted in the subsequent days of the observation. It should be noted that the clinical signs of intoxication, expressed in the condition of the animals, in the behavioral reactions, and in the pathological manifestations did not differ by gender.

Study results of acute toxicity after the intravenous administration in the laboratory rats with the determination of the median lethal dose, required in the further studies of an antiarrhythmic activity, are shown in Table 2.

**Table 2:** Indicators of lethal doses in the acute toxicity study of the piperidine derivatives via the intravenous administration route.

Compound/reference drug	LD_16_ (mg/kg)	LD_50_ (mg/kg)	LD_84_ (mg/kg)
LAS-286	218.6	238.4±11.64*^#^	259.4
LAS-294	83.3	94.7±6.9^#^	107.6
Procainamide	-	95±16.9	-
Allapinin	-	7.6 ±1.47	-

Data reported as means±SE (n=6). *P<0.001 compared to procainamide; ^#^P<0.001 compared to allapinin (*t*-test).

A statistically significant increase in the LD_50_ values of compound LAS-286 was established, exceeding those of procainamide and allapinin by 2.5 and 31 times, respectively. According to the data obtained, LAS-294 demonstrated greater toxicity than LAS-286. However, the LD_50_ value was significantly higher than allapinin by 12.5 times. In accordance with the OECD classification, the substances under study belong to hazard Class 3 (34).

With the intravenous administration of low dosages for 10-15 min, a decrease in the motor and exploratory activity was observed without the manifestation of inhibition of the basic physiological functions. In the next 10-15 min after the rapid recovery, the animals of the experimental group did not differ from the intact ones. The intoxication symptoms, when moderate dosages were administered, developed in the first minute after the administration and were accompanied by depression of the central nervous system, the development of clonic-tonic convulsions, followed by adynamia, respiratory depression, and the subsequent death of a number of the experimental animals in 20-30 s. The administration of the maximum dosages led to severe disturbances in the respiratory (gasping breathing) and cardiac activity (tachycardia, pallor of the mucous membranes and skin), developing from the moment of the administration or from the first seconds after completion of the administration for 5-10 s, where convulsive twitching of the muscles of the main groups also occurred.

### Evaluation of a local anesthetic effect

**Figure 2:**
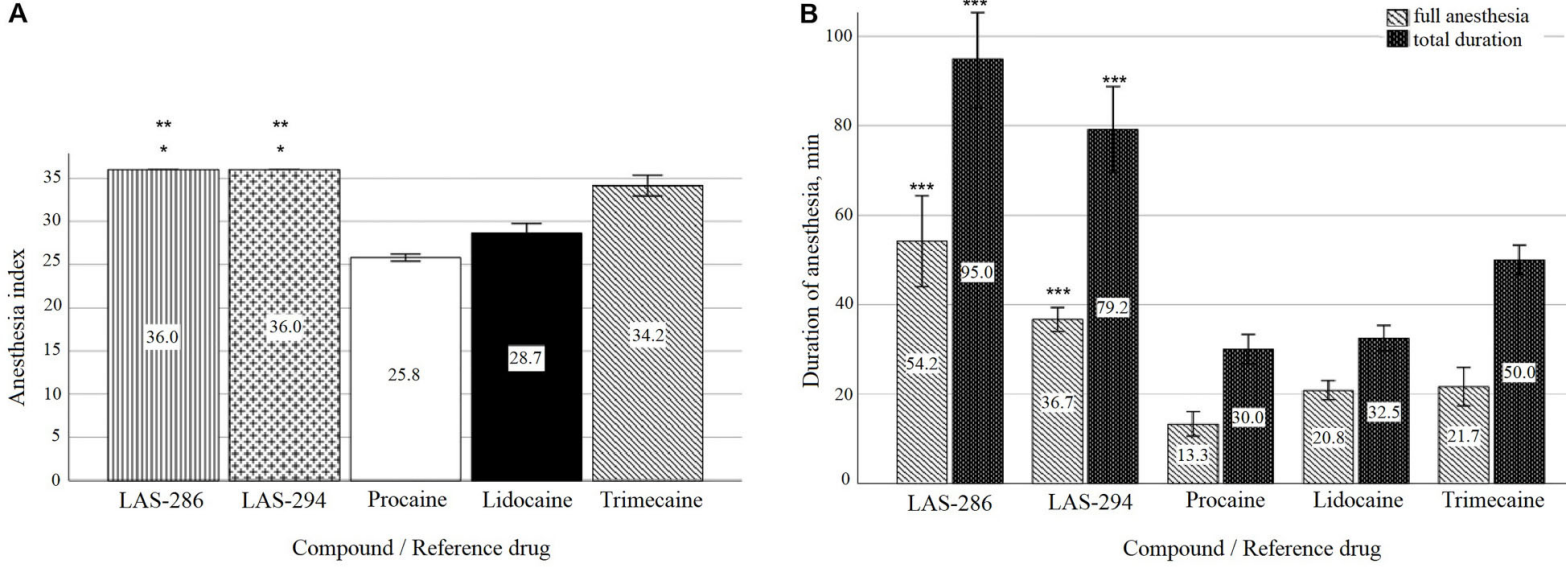
Indicators of the local anesthetic activity of the 0.5% solutions during infiltration anesthesia. Data are reported as means±SD (n=6). **A**, Anesthesia index (max-36). *P<0.05 compared to trimecaine, **P<0.001 compared to procaine and lidocaine (ANOVA). **B**, Duration of anesthesia. ***P<0.001 compared to all reference drugs (ANOVA).

The results of the local anesthetic activity of the new piperidine derivatives during the infiltration anesthesia are presented in Figure 2.

Statistically significant differences were observed in the anesthesia index, duration of full anesthesia, and total action of LAS-286, LAS-294, and reference drugs. A comparative analysis of the results has shown that the anesthesia index of tested compounds reached its maximum value and was significantly higher than that of the reference preparations, which indicated the presence of pronounced local anesthetic activity.

In terms of full anesthesia duration, LAS-286 significantly exceeded the clinically active comparison preparations: procaine by 4 times, lidocaine by 2.6 times, and trimecaine by 2.5 times. According to the presented results, the studied piperidine derivative acted longer than all the reference preparations, and the mean total duration of anesthesia was 95 min. The duration of both the full and the total duration of local anesthesia of LAS-294 was less prolonged than LAS-286, but significantly exceeded the comparison drugs. Thus, the duration of full anesthesia exceeded the appropriate indicator of the most active reference drug trimecaine by 15 min. The total duration of the local anesthetic effect of LAS-294 was more than 2 times higher than that of lidocaine and procaine, and its duration was 29.2 min longer than trimecaine.

In the performed in-depth study experiments, the following parameters were determined: the rate of anesthesia onset, the complete anesthesia duration, and the total duration. As shown in Table 3, all compounds showed a local anesthetic activity, with varying degrees of severity.

**Table 3:** Local anesthetic activity of the active compounds and reference preparations during the infiltration anesthesia of the abdominal wall in a rabbit (concentration of 0.5%).

Compounds/reference drugs	Anesthesia onset rate (min)	Duration of the complete anesthesia (min)	Total duration of anesthesia (min)
LAS-286	3.0±0	0	85.0±3.2**
LAS-294	15.0±1.8*	0	60.0±4.8*** ^#^
Procaine	3.0±0	0	23.8±1.5
Lidocaine	3.0±0	0	47.5±1.1
Trimecaine	3.0±0	5.0±0	86.6±2.1

Data reported as means±SE (n=6). *P<0.001 compared to reference drugs, **P<0.001 compared to procaine and lidocaine, ***P<0.001 compared to procaine and trimecaine, ^#^P<0.05 compared to lidocaine (*t*-test).

During the experiments, the duration of the infiltration anesthesia of LAS-286 exceeded that of all the studied compounds, procaine and lidocaine, and was comparable to trimecaine. The depth of anesthesia averaged 47%, and the rate of onset of anesthesia was the same as with the reference preparations.

LAS-294 was inferior to LAS-286 and trimecaine in terms of the duration of action, but at the same time it acted longer than procaine and lidocaine by 2.5 and 1.3 times, respectively. This piperidine derivative had a longer latency period compared with the others.

Thus, the in-depth studies of the local anesthetic effect by infiltration of the anterior abdominal wall of a rabbit confirmed the presence of an effect in LAS-286 and LAS-294 with the achievement of a depth of anesthesia in the laboratory animals of the experimental group of 33 to 47%, but without the onset of complete 100% anesthesia. There was some advantage in the duration of the induced anesthesia for LAS-286. Thus, based on the results of a series of experiments, it can be concluded that LAS-286 was the most active compound with local anesthetic activity during the infiltration anesthesia.

### Evaluation of an antiarrhythmic effect

The intravenous administration of aconitine at the dosage of 12 µg/kg caused the development of mixed arrhythmia in all cases of the control group. A pronounced rhythm disturbance in the form of paroxysmal tachycardia appeared already in the first minute after the administration of the aconitine solution, progressing to critical ventricular fibrillation in all experimental animals, and led to death in a short period of time in the first 10 min of the observation. The solutions of LAS-286 and LAS-294 substances had been administered over a wide range of doses depending on the efficacy detected. In effective doses, the solutions of these compounds prevented the development of arrhythmia and reduced mortality. A comparative study of the antiarrhythmic effectiveness of the studied compounds in a series of experiments on the aconitine model is presented in Table 4.

**Table 4:** Comparative antiarrhythmic activity of the compounds LAS-286 and LAS-294 with modern antiarrhythmic drugs on the model of aconitine-induced arrhythmia (experiments on rats).

Compounds/reference drugs	Aconitine (12 µg/kg, *iv*)						
	LD_50_ (mg/kg, *iv*)	Dose (mg/kg, *iv*)	Total(n)	With arrhythmia (n)	Without arrhythmia (n)	Antiarrhythmic effect (%)	Antiarrhythmic index LD_50_/ED_50_
Control	-	H_2_O, 0.2 mL	10	10	-	-	-
LAS-286	238.4	0.5	10	8	2	20	335.8
		1.0	10	6	4	40	
		5.0	10	8	2	20	
		10.0	10	9	1	10	
		15.0	10	10	-	-	
LAS-294	94.7	0.05	10	5	5	50	242.8
		0.1	10	1	9	90	
		0.5	10	6	4	40	
		1.0	10	7	3	30	
		5.0	10	10	-	-	
		10.0	10	10	-	-	
Procainamide	95.0	12.0	10	2	8	80	6.1
		20.0	10	5	5	50	
Allapinin	7.6	0.05	10	1	9	90	15.2

**Figure 3:**
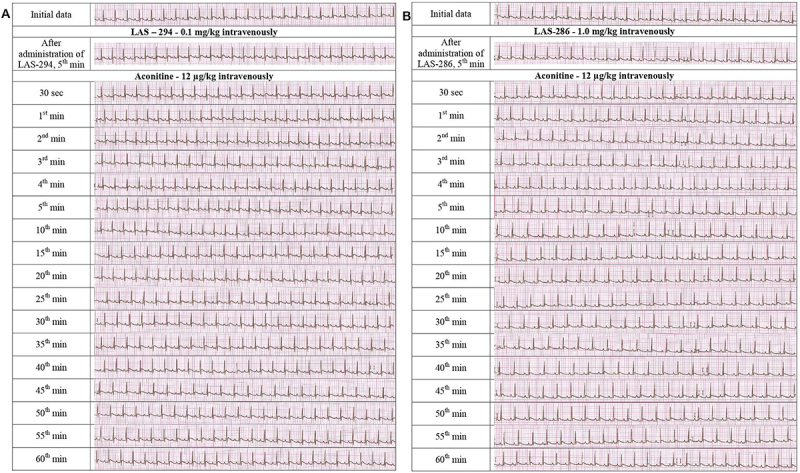
ECG dynamics with the intravenous administration of 0.1 mg/kg LAS-294 (**A**) and 1.0 mg/kg LAS-286 (**B**) on the aconitine model of arrhythmia (V=50 mm/s).

The studied piperidine derivatives showed their effectiveness to varying degrees. The compound LAS-294 showed a more pronounced antiarrhythmic activity in the development of aconitine-induced arrhythmia, where at the dosage of 0.1 mg/kg this substance prevented the development of aconitine arrhythmia in 90% of rats. A stable rhythm control after the aconitine administration was observed throughout the experiment without lethal outcomes (Figure 3). A similar effect was observed when using allapinin at the dosage of 0.05 mg/kg, similar to procainamide at the dose of 12.0 mg/kg. In the remaining groups, the effective antiarrhythmic doses of LAS-294 ranged from 0.05 mg/kg (50%) to 0.5 mg/kg (40%). Under similar experimental conditions, LAS-286 was inferior in activity to the comparison preparations and exhibited a moderate suppressive antiarrhythmic activity (40%) at the dosage of 1.0 mg/kg. It is worth noting that the two piperidine derivatives acted in low dosages, while increasing the dosage led to a decrease in their antiarrhythmic activity.

The median effective dose of LAS-294 was 0.39 mg/kg. A comparison of the median effective antiarrhythmic doses of LAS-294 with the known antiarrhythmic preparations showed its antiarrhythmic activity was 40 times superior than procainamide and 1.3 times superior than allapinin. To assess the breadth of the therapeutic action in case of arrhythmia, the antiarrhythmic index was calculated and compared with the standard antiarrhythmics. LAS-294 significantly exceeded procainamide and allapinin in terms of the breadth of the pharmacological action by 39.8 and 16 times, respectively, which indicated its safety in terms of practical use.

Despite the lower efficiency, the ED_50_ of LAS-286 was 0.71 mg/kg, which is significantly higher than that of procainamide by 22 times, being close to allapinin. Also, our experiments revealed the advantages of this substance in terms of the therapeutic dose range, surpassing procainamide by 55 times and allapinin by 22 times, which indicates a high degree of safety.

Thus, LAS-294 exhibited a high antiarrhythmic activity when administered intravenously against the background of the aconitine arrhythmia in laboratory rats, surpassing the standard antiarrhythmic drugs. This compound at the dosage of 0.05-0.1 mg/kg completely eliminated the arrhythmia and restored the sinus rhythm in 50-90% of animals. Despite the low effectiveness, the study results indicated the high safety of LAS-286. These compounds, unlike most antiarrhythmic drugs, did not have an arrhythmogenic effect, which was confirmed by the ECG indicators in all series of experiments. In accordance with the results obtained, it can be assumed that LAS-294 and LAS-286 are able to act on the sodium channels and reduce the increased flow of the Na^+^ ions through the membrane due to the disruption of the functional activity of the voltage-gated sodium channels, caused by the introduction of aconitine. Consequently, the intravenous administration of the studied compound presumably leads to a pronounced membrane-stabilizing effect, causing the antiarrhythmic effect characteristic of the Class 1 antiarrhythmic drugs (35).

### Molecular docking results

Based on the results of experimental studies of local anesthetic and antiarrhythmic activity, macromolecules of sodium channels Na_v_1.4 (PDB ID: 6AGF) and Na_v_1.5 (PDB ID: 7DTC) were selected as targets for molecular docking. The docking results of the tested compounds are reported as binding energy (in kcal/mol) and are presented in Table 5. In the docking study of the target compounds LAS-286 and LAS-294, the two compounds had similar docking values, which slightly exceeded the performance of the reference drug lidocaine.

**Table 5:** Results of the molecular docking studies.

Compounds	Docking force (kcal/mol)	
	Na_v_1.4	Na_v_1.5
LAS-286	-7.2	-7.2
LAS-294	-7.3	-7.0
Lidocaine	-5.9	-6.4

**Figure 4:**
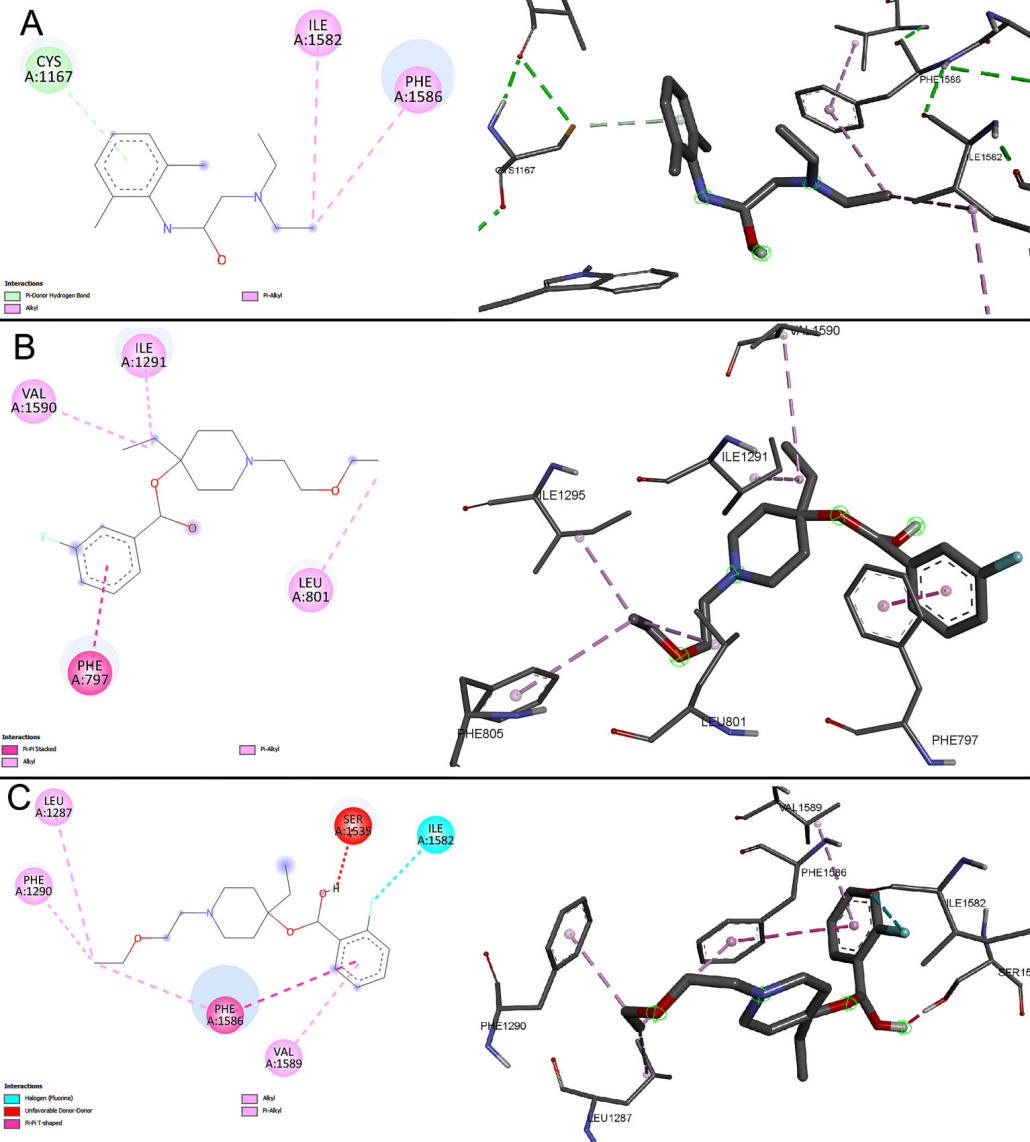
Docking of the Na_v_1.4 macromolecule and the studied ligands. **A**, lidocaine; **B**, LAS-286; **C**, LAS-294.

**Figure 5:**
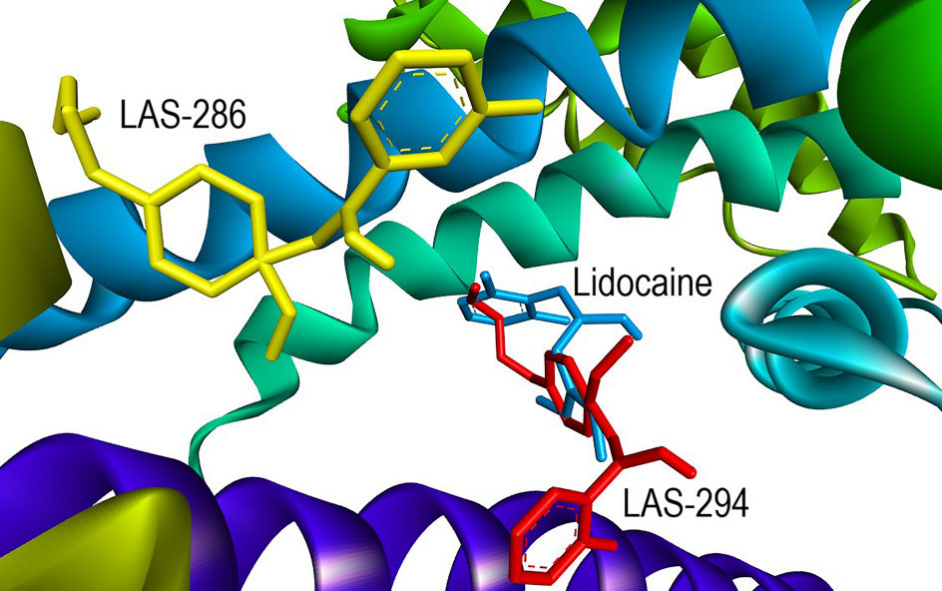
Ligand positions in the Na_v_1.4 macromolecule.

During the docking of the Na_v_1.4 macromolecule with three different ligands (lidocaine, LAS-286, and LAS-294), significant interactions were observed, as depicted in Figures 4 and 5. Docking results of the LAS-286 ligand with the Na_v_1.4 protein revealed distinct interactions compared to lidocaine and amino acids in the protein's active site. The aromatic ring of LAS-286 demonstrated interaction with the amino acid PHE A:797 through a Pi-Pi Stacked bond. The ethynyl group, in turn, formed bonds with the amino acids ILE A:1291 and VAL A:1590 through alkyl and Pi-alkyl interactions, respectively. Other amino acids (ILE A:1295, LEU A:801, and PHE A:805) in the active site were linked to the end of the ethoxyethyl: the first two via Pi-alkyl bonds and the latter via alkyl.

Docking results of the LAS-294 ligand in Na_v_1.4 showed similar interactions to lidocaine and analogous amino acids in the protein's active site. The benzene ring of LAS-294 interacted with the amino acid PHE A:1586 through a Pi-Pi T-shaped bond and with the amino acid VAL A:1589 through an alkyl bond, whereas lidocaine formed a Pi-alkyl bond with the same amino acid. The fluorine atom in LAS-294 interacted with ILE A:1582 via a halogen (fluorine) bond, while lidocaine formed an alkyl bond with the same amino acid. Like LAS-286, LAS-294 interacted with three amino acids at the free end of the ethoxyethyl. Two of them, LEU A:1287 and PHE A:1290, were linked via an alkyl bond, while PHE A:1586, interacting with the benzene ring, was linked via a Pi-alkyl bond. However, an undesirable donor-donor interaction between LAS-294 and SER A:1535 was observed.

**Figure 6:**
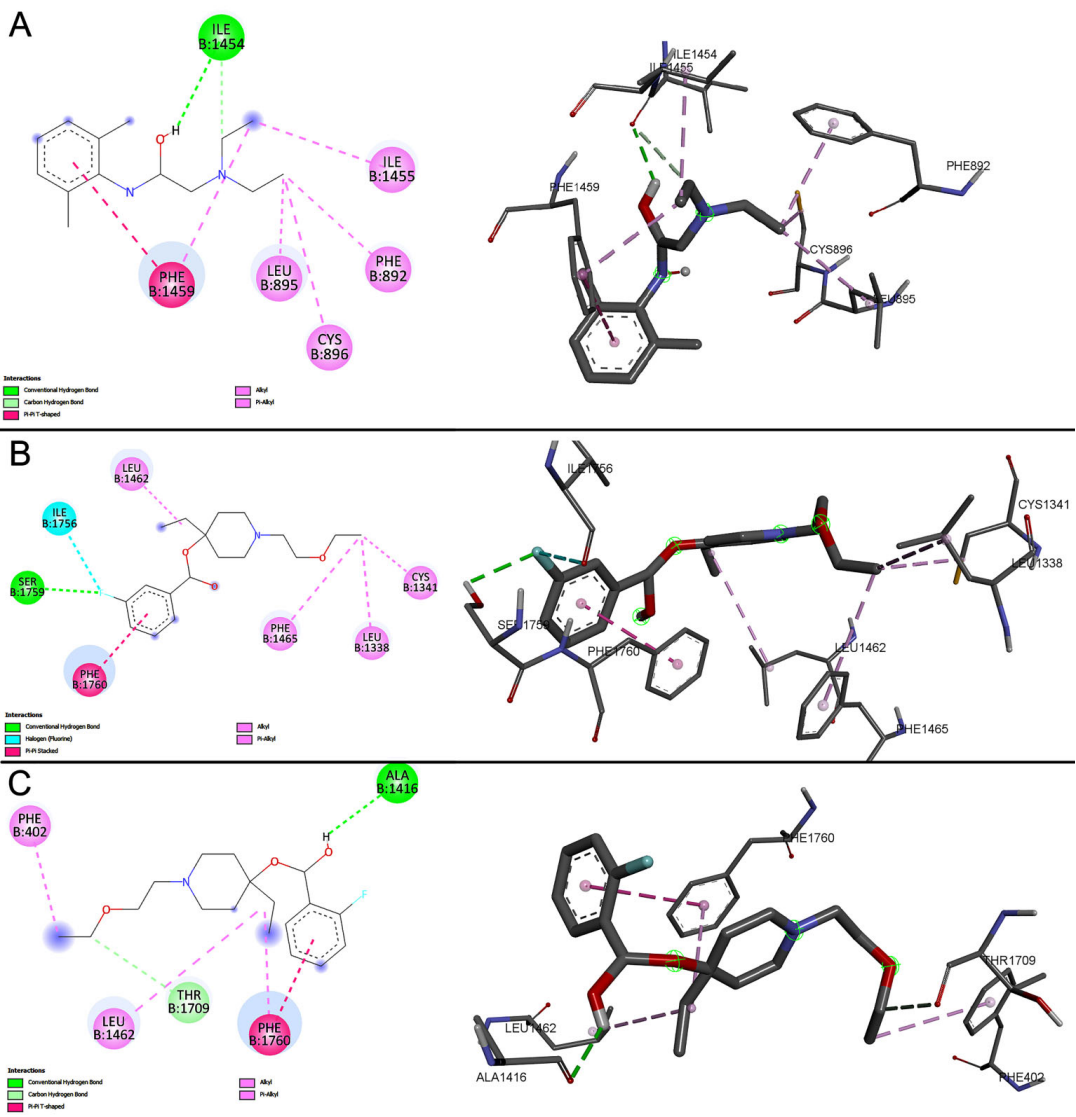
Docking of the Na_v_1.5 macromolecule and the studied ligands. **A**, lidocaine; **B**, LAS-286; **C**, LAS-294.

**Figure 7:**
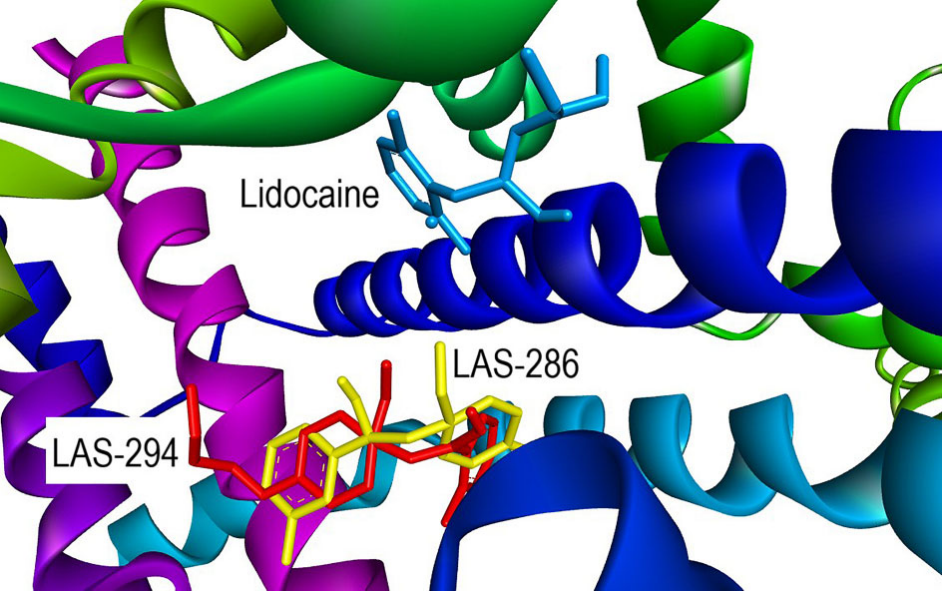
Ligand positions in the Na_v_1.5 macromolecule.

The docking results of the Na_v_1.5 macromolecule with three ligands (lidocaine, LAS-286, and LAS-294) showed that the investigated ligands differed from lidocaine in chemical interactions and amino acids (Figures 6 and 7). Lidocaine was bound to PHE B:1459 through a Pi-Pi T-shaped bond in the aromatic ring. Additionally, PHE B:1459, as well as the amino acids ILE B:1455, and LEU B:895, interacted with the free end of lidocaine's diethylamino group via Pi-alkyl bonds. Also, ILE B:1454, PHE B:892, and CYS B:896 were bound by conventional hydrogen bonds and two amino acids by alkyl bonds, respectively.

In the case of LAS-286 and LAS-294, the aromatic rings of both interacted with the amino acid PHE B:1760, but LAS-286 was bound via Pi-Pi stacked bond, while LAS-294 was bound via Pi-Pi T-shaped bond. In LAS-294, PHE B:1760 was additionally bound to ethynyl via a Pi-alkyl bond. Both ligands were bound to LEU B:1462 via Pi-alkyl bonds in ethynyl.

Other interactions differed from each other. LAS-286 was bound via a halogen (fluorine) bond to ILE B:1756 and by a conventional hydrogen bond to SER B:1759 in the same region. Also, there were alkyl bonds in both ligands in the ethoxyethyl region: CYS B:1341, LEU B:1338, PHE B:1465 for LAS-286, and PHE B:402 for LAS-294.

The ligands - lidocaine, LAS-286, and LAS-294 - were bound to macromolecules with values of -5.9, -7.2, and -7.3 in the case of Na_v_1.4 and -6.4, -7.2, -7.0 in the case of Na_v_1.5, respectively.

In its integrity, the results provide important scientific insights into potential molecular interactions, which may have implications for the future development of medicinal preparations. The molecular docking of the current study for the piperidine derivatives LAS-286 and LAS-294 showed the presence of a strong bond when docked with sodium channel macromolecules. The pronounced local anesthetic and antiarrhythmic activities of the studied compounds observed during the experiments were supported by the study of molecular docking and indicate a high therapeutic potential for further research.

## Supplementary Material

Click here to view [pdf].

## References

[1] Yu S, Wang B, Zhang J, Fang K (2019). The development of local anesthetics and their applications beyond anesthesia. Int J Clin Exp Med.

[2] Noyev A, Kuznetsov N, Korenev G, Morozova N, Vasil'ev Y, Suvorov N (2022). A novel photoswitchable azobenzene-containing local anesthetic ethercaine with light-controlled biological activity *in vivo*. Int J Mol Sci.

[3] Woolf CJ (2010). What is this thing called pain?. J Clin Invest.

[4] Zhou S, Huang G, Chen G (2019). Synthesis and biological activities of local anesthetics. RSC Adv.

[5] Basbaum AI, Bautista DM, Scherrer G, Julius D (2009). Cellular and molecular mechanisms of pain. Cell.

[6] Cherobin ACFP, Tavares GT (2020). Safety of local anesthetics. An Bras Dermatol.

[7] Lirk P, Hollmann MW, Strichartz G (2018). The science of local anesthesia: basic research, clinical application, and future directions. Anesth Analg.

[8] Chitilian HV, Eckenhoff RG, Raines DE (2013). Anesthetic drug development: Novel drugs and new approaches. Surg Neurol Int.

[9] Scholz A (2002). Mechanisms of (local) anaesthetics on voltage-gated sodium and other ion channels. Br J Anaesth.

[B10] Nattel S, Duker G, Carlsson L (2008). Model systems for the discovery and development of antiarrhythmic drugs. Prog Biophys Mol Biol.

[11] Nguyen PT, DeMarco KR, Vorobyov I, Clancy CE, Yarov-Yarovoy V (2019). Structural basis for antiarrhythmic drug interactions with the human cardiac sodium channel. Proc Natl Acad Sci USA.

[12] Goel P, Alam O, Naim MJ, Nawaz F, Iqbal M, Alam MI (2018). Recent advancement of piperidine moiety in treatment of cancer-a review. Eur J Med Chem.

[13] Holtschulte C, Börgel F, Westphälinger S, Schepmann D, Civenni G, Laurini E (2022). Synthesis of aminoethyl-substituted piperidine derivatives as σ receptor ligands with antiproliferative properties. ChemMedChem.

[14] Liu GQ, Opatz T (2018). Recent advances in the synthesis of piperidines: functionalization of preexisting ring systems. Adv Heterocycl Chem.

[15] Martins ML, Eckert J, Jacobsen H, Dos Santos ÉC, Ignazzi R, de Araujo DR (2017). Probing the dynamics of complexed local anesthetics via neutron scattering spectroscopy and DFT calculations. Int J Pharm.

[16] Vasilyuk AA, Kozlovsky VI (2021). Promising directions for the application of piperidine derivatives as structural components of neurotropic. Vestnik VGMU.

[17] Kemelbekov US, Hagenbach A, Lentz D, Imachova ShO, Pichkhadze GM, Rustembekov ZhI (2010). Pharmacology and structures of the free base of the anaesthetickazcaine and its complex with β-cyclodextrin. J Incl Phenom Macrocycl Chem.

[B18] Kadyrova DM, Pichkhadze GM, Pralyev KD, Yu VK (2010). Kazсkain - perspective native local anaesthetic [in Russian]. Vestnik KazNMU.

[B19] Praliev KD, Isin Zh, Yu VK, Tarakov SA, Bosyakov YuG, Utepbergenova RK (1996). Hydrochloride of 1-(2-ethoxyethyl)-4-ethynyl-4-benzoyloxy-piperidine possesses anesthetic activity. Patent Ru.

[20] Praliyev KD, Isin ZhI, Yu VK, Tarakov SA, Bosyakov YuG, Utepbergenova RK (1996). Kazcaine-1-(2-ethoxyethyl)-4-ethynylpiperidin-4-yl benzoate hydrochloride. https://kzpatents.com/5-3137-gidrohlorid-1-2-etoksietil-4-etinil-4-benzoiloksipiperidina-obladayushhijj-mestnoanestiziruyushhejj-aktivnostyu.html?ysclid=lwxwz43imn972470988.

[21] Granger B, Albu S (2005). The haloperidol story. Ann Clin Psychiatry.

[22] Issayeva UB, Akhmetova GS, Datkhayev UM, Omyrzakov MT, Praliyev KD, Ross SA (2019). The search for biologically active compounds in the series of N-ethoxyethylpiperidine derivatives. Eurasian Chem Technol J.

[23] Dolomanov OV, Bourhis LJ, Gildea RJ, Howard JAK, Puschmann H (2009). OLEX2: A complete structure solution, refinement and analysis program. J Appl Cryst.

[24] Farrugia LJ (1997). ORTEP-3 for Windows - a version of ORTEP-III with a Graphical User Interface (GUI). J Appl Crystallogr.

[25] Spek AL (2003). Single-crystal structure validation with the program PLATON. J Appl Crystallogr.

[26] Daina A, Michielin O, Zoete V (2019). SwissTargetPrediction: updated data and new features for efficient prediction of protein targets of small molecules. Nucleic Acids Res.

[27] Filimonov DA, Lagunin A, Gloriozova T, Rudik AV, Druzhilovskii DS, Pogodin PV (2014). Prediction of the biological activity spectra of organic compounds using the pass online web resource. Chem Heterocycl Comp.

[28] Mironov AN (2012). Guidelines for conducting preclinical studies of medicines [in Russian]. Part one. Grif & Co: Moscow, Russia.

[29] Bulbring E, Wajda I (1945). Biological comparison of local anaesthetics. J Pharmacol Exp Ther.

[30] Kuzenbayeva RS, Rakhimov KD, Shin SN, Chukanova GN (2000). Methodological guide: Preclinical study of local anesthetic activity of new biologically active substances [in Russian, in print only].

[31] Nattel S, Duker G, Carlsson L (2008). Model systems for the discovery and development of antiarrhythmic drugs. Prog Biophys Mol Biol.

[32] Randhawa MA (2009). Calculation of LD50 values from the method of Miller and Tainter, 1944. J Ayub Med Coll Abbottabad.

[33] Imanbekov K, Beketov K, Yu V, Praliyev K (2011). Crystal structure of 1-(2-ethoxyethyl)-4-ethynyl-4-benzoyloxypiperidine hydrochloride, C18H24ClNO3. Z Kristallogr NCS 226.

[34] Berezovskaya IV (2003). Classification of substances with respect to acute toxicity for parenteral administration. Pharm Chem J.

[35] Lei M, Wu L, Terrar DA, Huang CL (2018). Modernized classification of cardiac antiarrhythmic drugs. Circulation.

