# Supplementary material for: Experimental study of local anesthetic and antiarrhythmic activities of fluorinated ethynylpiperidine derivatives
Source: Braz J Med Biol Res. 2024 Jul 29;57:e13429. doi: 10.1590/1414-431X2024e13429 (PMC11290815; doi:10.1590/1414-431X2024e13429)
Supplement: Supplementary file 1 [file 1414-431X-bjmbr-57-e13429-suppl.pdf]

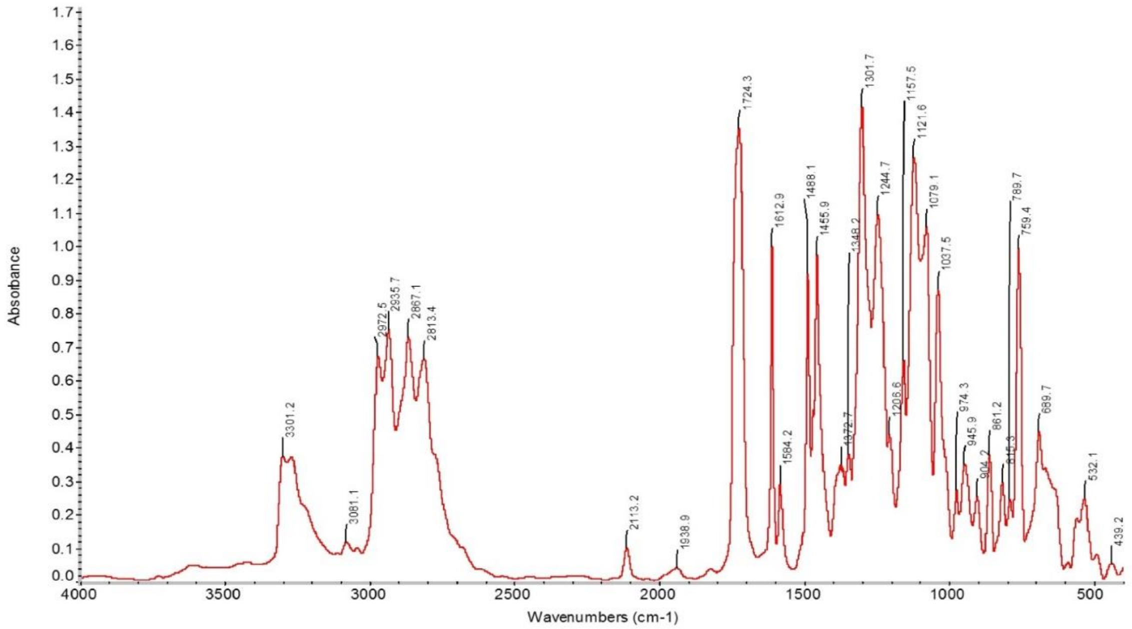

Figure S1. Infrared spectrum (cm^{-1}) of 1-(2-ethoxyethyl)-4-ethynylpiperidin-4-yl *o*-fluorobenzoate (LAS-294 base): 1724.3 (C=O); 1301.7 (C-F) ; 1121.6 (C-O-C); 1612.9 (C=C aryl.); 2113.2 (C≡C); 3301.2 (≡C-H).

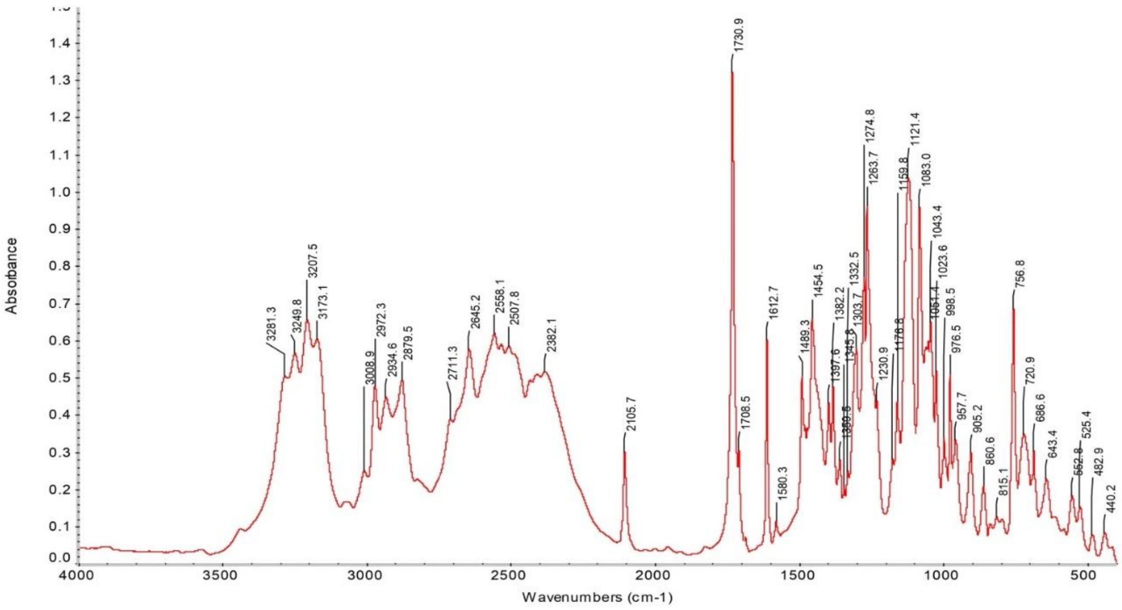

Figure S2. Infrared spectrum of 1-(2-ethoxyethyl)-4-ethynylpiperidin-4-yl *o*-fluorobenzoate hydrochloride (LAS-294): 1730.9 (C=O); 1263.7 (C-F); 1176.8 (C-O-C); 1612.7 (C=C aryl.); 2105.7 (C≡C); 3249.8 (≡C-H).

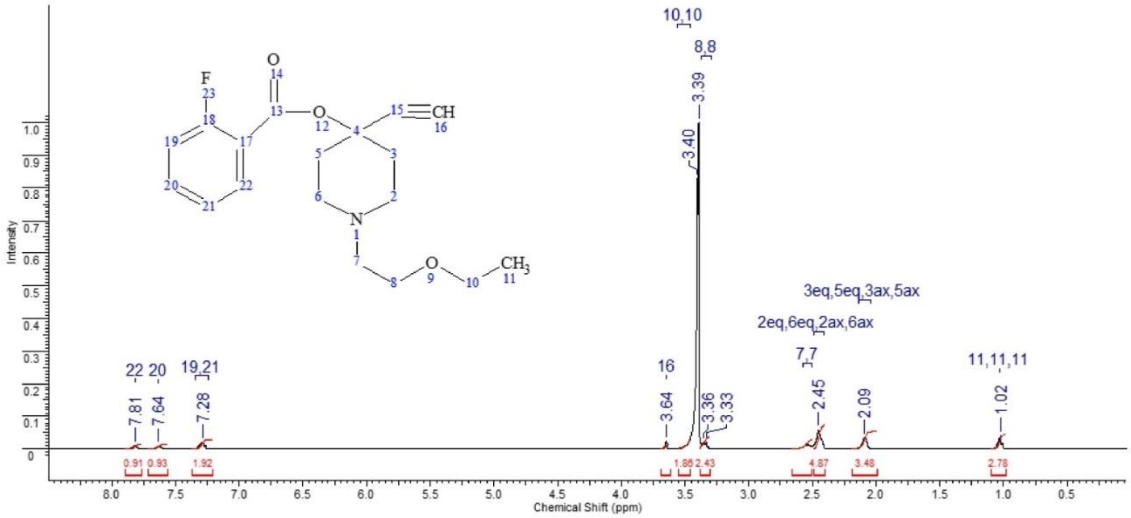

Figure S3. ^{1}H NMR spectrum of 1-(2-ethoxyethyl)-4-ethynylpiperidin-4-yl *o*-fluorobenzoate (LAS-294 base) in DMSO-d_6, δ, ppm, (*J*, Гц): 1.00–1.05 (3H, м, $H^{11,11,11}$), 2.09 (4H, widened s, $H^{3a,5a,3e,5e}$), 2.45 (4H, уш. с, $H^{2a,6a,2e,6e}$), 2.54 (2H, widened s, $H^{7,7}$), 3.31–3.37 (2H, m, $H^{8,8}$), 3.45–3.54 (2H, m, $H^{10,10}$), 3.64 (1H, s, H^{16}), 7.26–7.31 (2H, m, $H^{19,21}$), 762–7.64 (1H, m, H^{20}), 7.80–7.83 (1H, m, H^{22}).

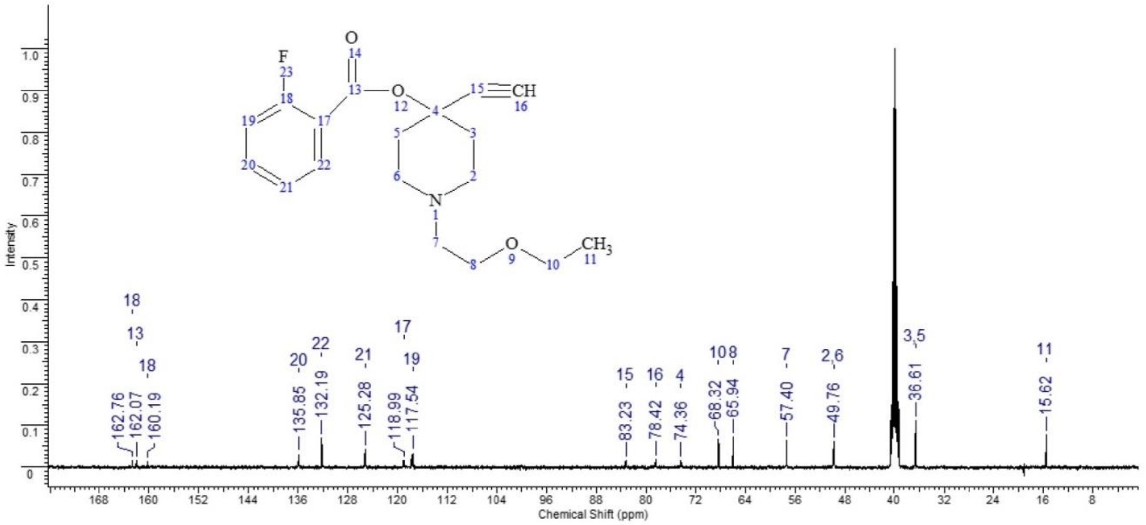

Figure S4. ^{13}C NMR spectrum of 1-(2-ethoxyethyl)-4-ethynylpiperidin-4-yl *o*-fluorobenzoate (LAS-294 base) in DMSO-d_6, δ, ppm: 15.62 (C^{11}), 36.61 ($C^{3,5}$), 49.76 ($C^{2,6}$), 57.40 (C^{7}), 65.94 (C^{8}), 68.32 (C^{10}), 74.36 (C^{4}), 78.42 (C^{16}), 83.23 (C^{15}), 117.54 и 117.76 (C^{19}), 118.99 и 119.09 (C^{17}), 125.28 (C^{21}), 132.19 (C^{22}), 135.85 (C^{20}), 160.19 и 162.76 (C^{18}), 162.07 (C^{13}).

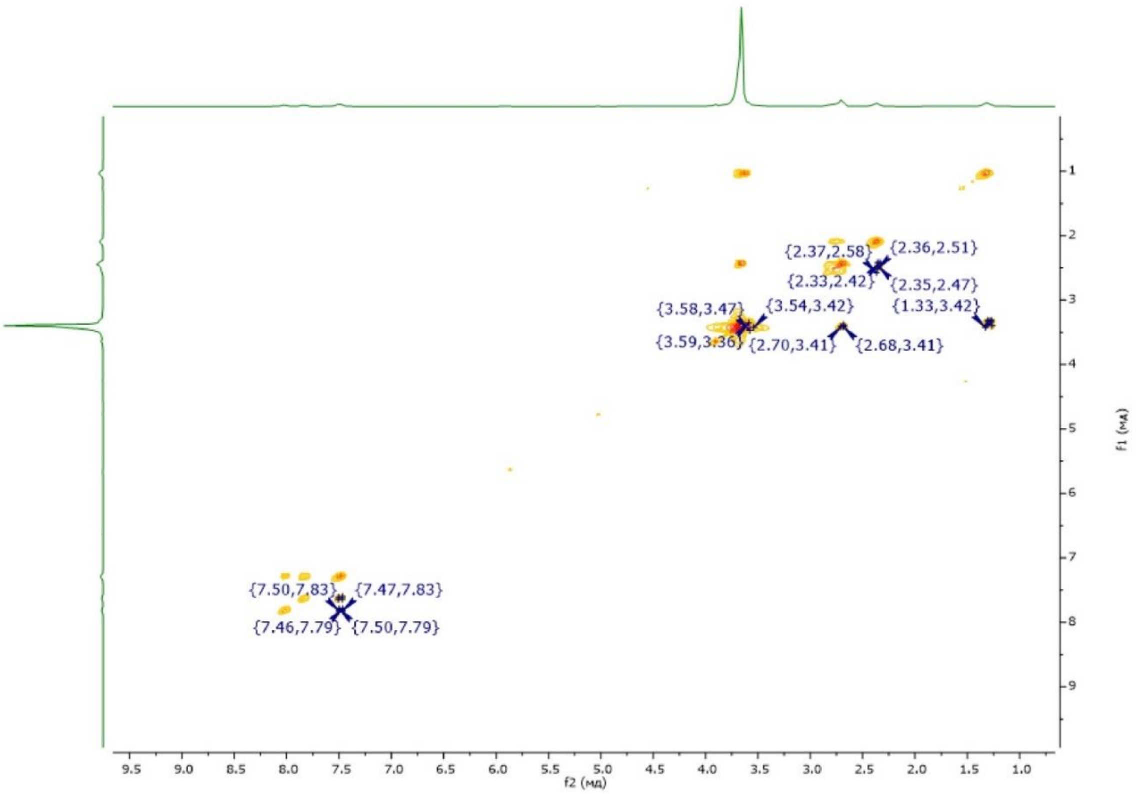

Figure S5. NMR spectrum COSY of 1-(2-ethoxyethyl)-4-ethynylpiperidin-4-yl *o*-fluorobenzoate (LAS-294 base) in DMSO-d$_6$: $H^{3,5}\rightarrow H^{2a,6a}$, $H^{11}\rightarrow H^{10}$, $H^{2a,6a}\rightarrow H^{10}$, $H^{19}\rightarrow H^{20}$, $H^{21}\rightarrow H^{22}$.

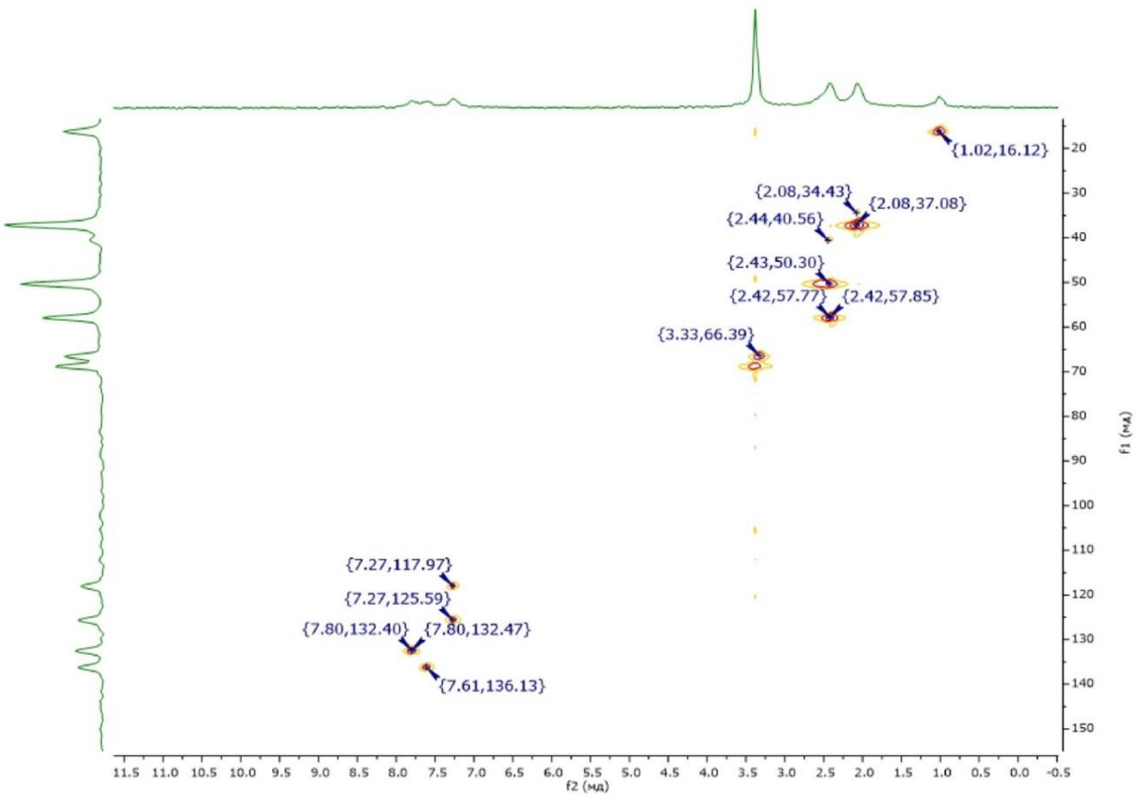

Figure S6. NMR spectrum HMQC of 1-(2-ethoxyethyl)-4-ethynylpiperidin-4-yl *o*-fluorobenzoate (LAS-294 base) in DMSO-d_6: H^{11}→C^{11}, $H^{3,5}$→$C^{3,5}$, $H^{2eq,6eq}$→$C^{2,6}$, H^7→C^7, H^8→C^8, H^{10}→C^{10}, H^{19}→C^{19}, H^{21}→C^{21}, H^{22}→C^{22}, H^{20}→C^{20}.

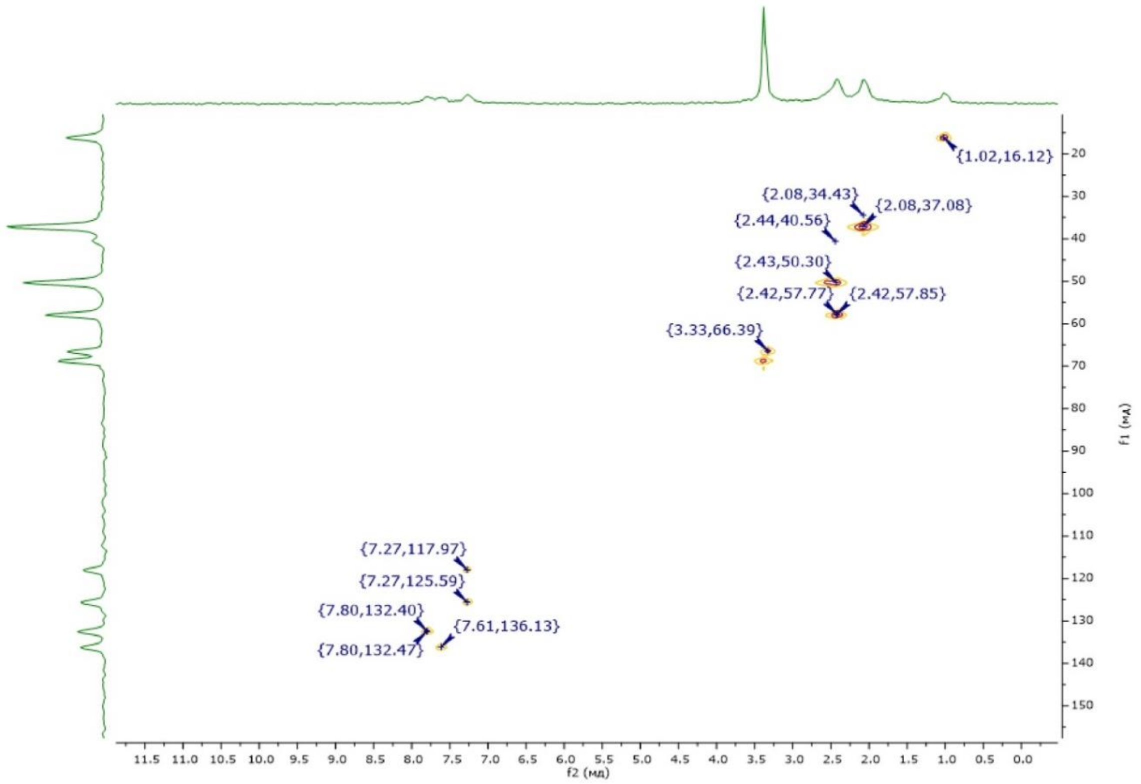

Figure S7. NMR spectrum HMBC of 1-(2-ethoxyethyl)-4-ethynylpiperidin-4-yl *o*-fluorobenzoate (LAS-294 base) in DMSO-d$_6$: $H^{11}\rightarrow C^8$; $H^{2,6}\rightarrow C^{10}$; $H^8\rightarrow C^{11}$, C^4; $H^{16}\rightarrow C^4$; $H^{19}\rightarrow C^{17}$, C^{21}; $H^{17}\rightarrow C^{19}$, C^{18}, C^{15}, C^{16}; $H^{20}\rightarrow C^{22}$; $H^{22}\rightarrow C^{20}$, C^{13}.

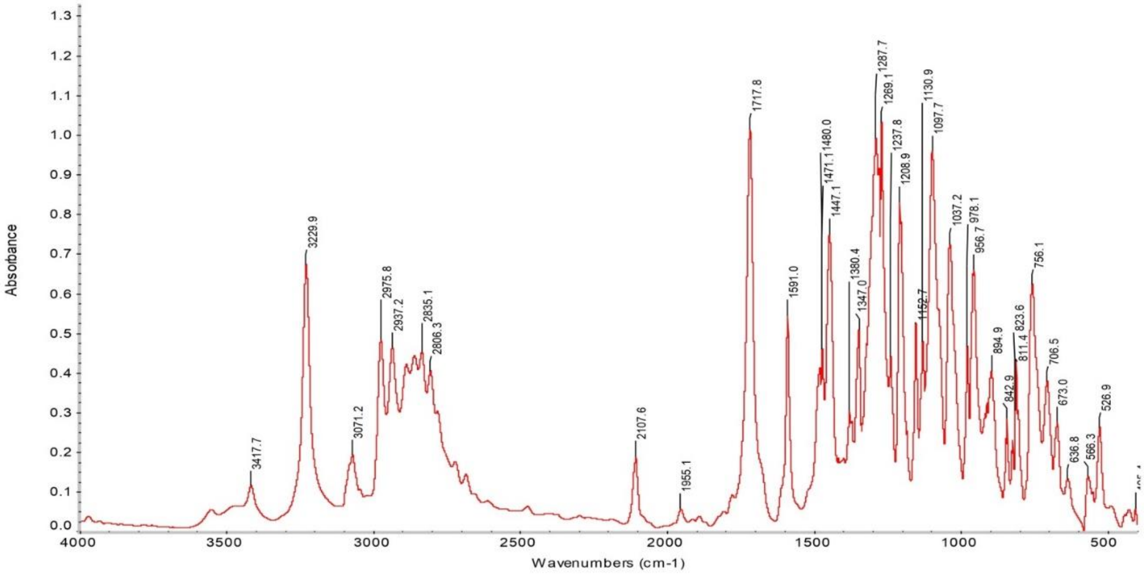

Figure S8. Infrared spectrum cm^{-1} of 1-(2-ethoxyethyl)-4-ethynylpiperidin-4-yl *m*-fluorobenzoate (LAS-286 base): 1717.8 (C=O); 1287.7 (C-F); 1097.7 (C-O-C); 1591.0 (C=C aryl.); 2107.6 (C≡C); 3229.9 (≡C-H).

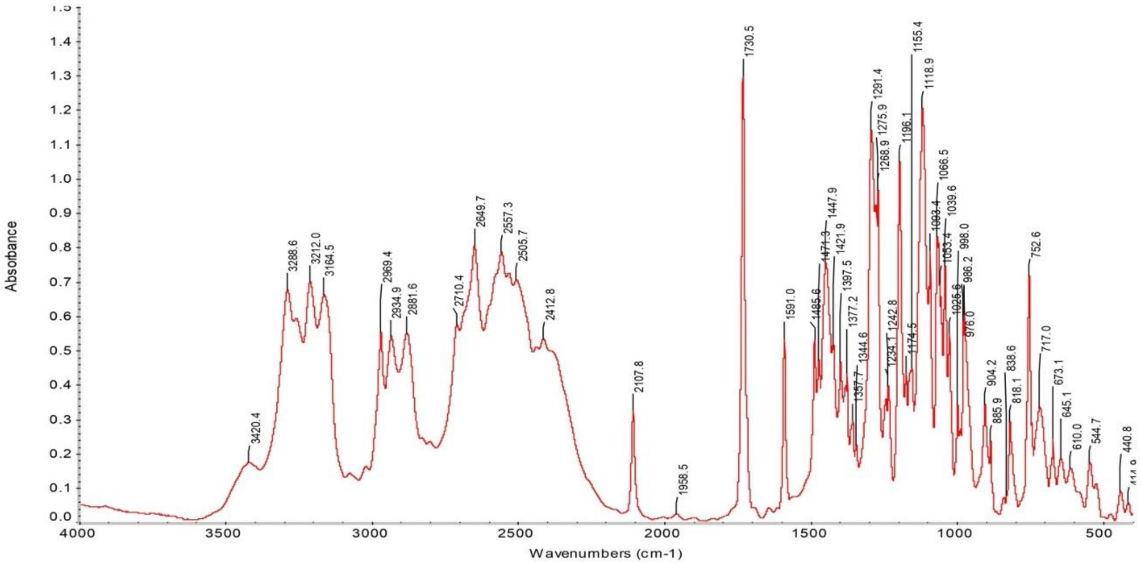

Figure S9. Infrared spectrum of 1-(2-ethoxyethyl)-4-ethynylpiperidin-4-yl *m*-fluorobenzoate hydrochloride (LAS-286): 1730.5 (C=O); 1291.4 (C-F); 1118.9 (C-O-C); 1591.0 (C=C aryl); 2107.8 (C≡C); 3288.6≡C-H).

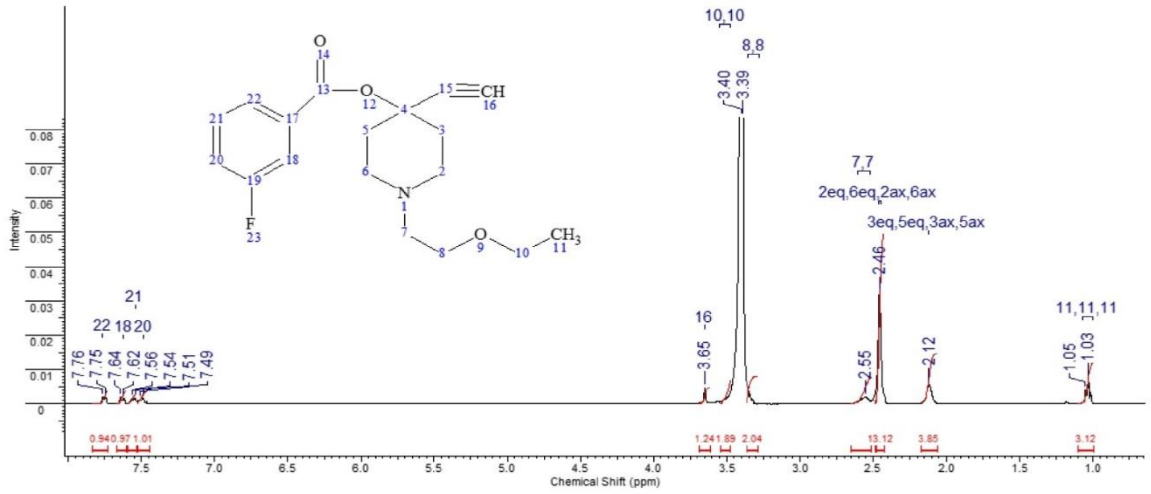

Figure S10. ^{1}H NMR spectrum of 1-(2-ethoxyethyl)-4-ethynylpiperidin-4-yl *m*-fluorobenzoate (LAS-286 base) in DMSO-d_6, δ, ppm, (*J*, Гц): 1.01–1.05 (3H, m, $H^{11,11,11}$), 2.12 (4H, widened s, $H^{3a,5a,3e,5e}$), 2.46 (4H, widened s, $H^{2a,6a,2e,6e}$), 2.55 (2H, widened s, $H^{7,7}$), 3.31–3.38 (2H, m, $H^{8,8}$), 3.47–3.59 (2H, m, $H^{10,10}$), 3.65 (1H, s, H^{16}), 7.45–7.52 (1H, m, H^{20}), 7.53–7.58 (1H, m, H^{21}), 7.62–7.64 (1H, m, H^{18}), 7.73–7.76 (1H, m, H^{22}).

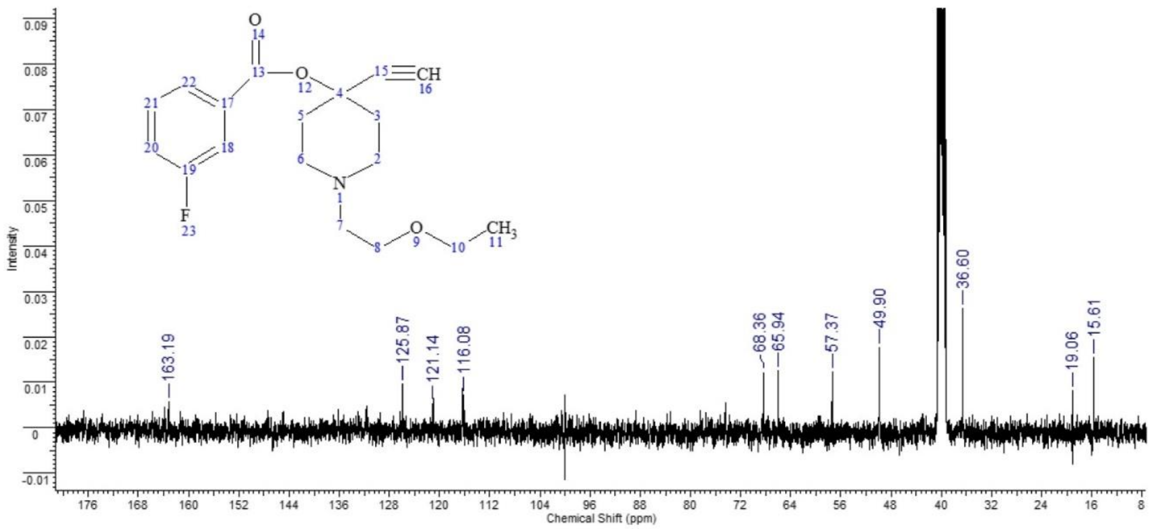

Figure S11. ^{13}C NMR spectrum of 1-(2-ethoxyethyl)-4-ethynylpiperidin-4-yl *m*-fluorobenzoate (LAS-286 base) in DMSO-d$_6$, δ, ppm: 15.61 (C^{11}), 36.60 (C^{3,5}), 49.90 (C^{2,6}), 57.37 (C^{7}), 65.94 (C^{8}), 68.36 (C^{10}), 116.08 (C^{18}), 121.14 (C^{20}), 125.87 (C^{22}), 163.19 (C^{13}).

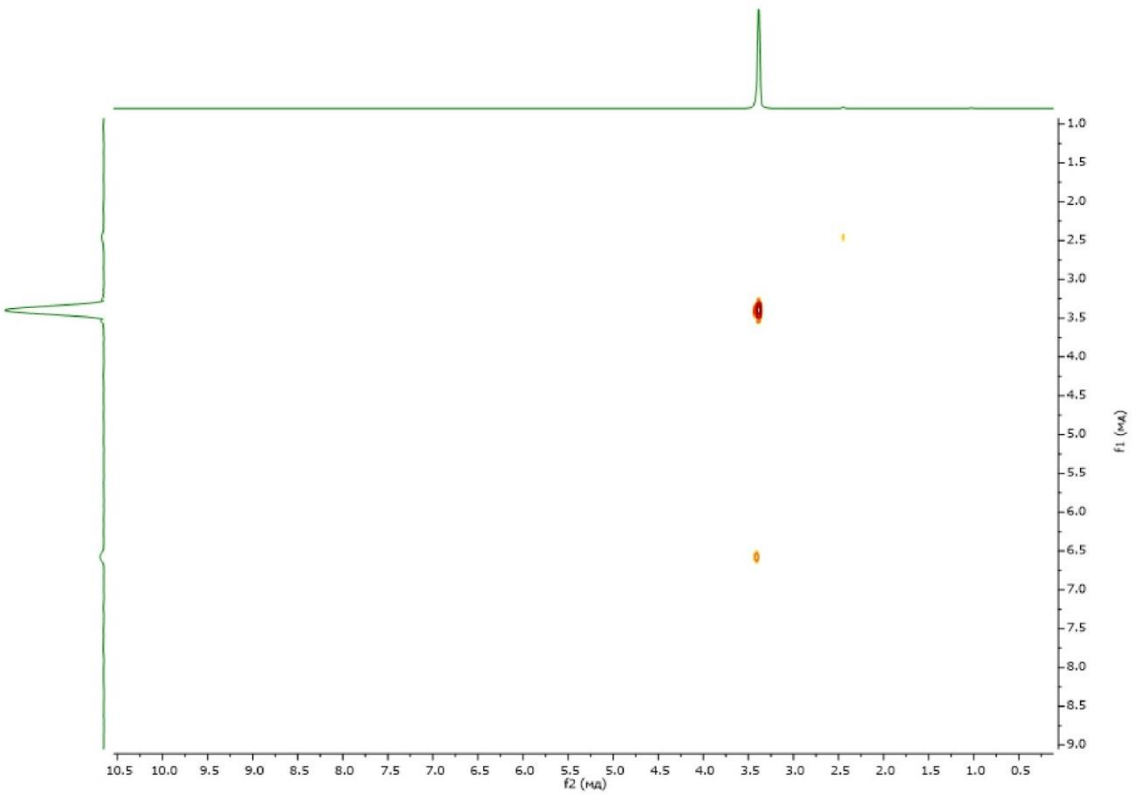

Figure S12. NMR spectrum COSY of 1-(2-ethoxyethyl)-4-ethynylpiperidin-4-yl *m*-fluorobenzoate (LAS-286 base) in DMSO-d_6: $H^{11} \rightarrow H^8$.

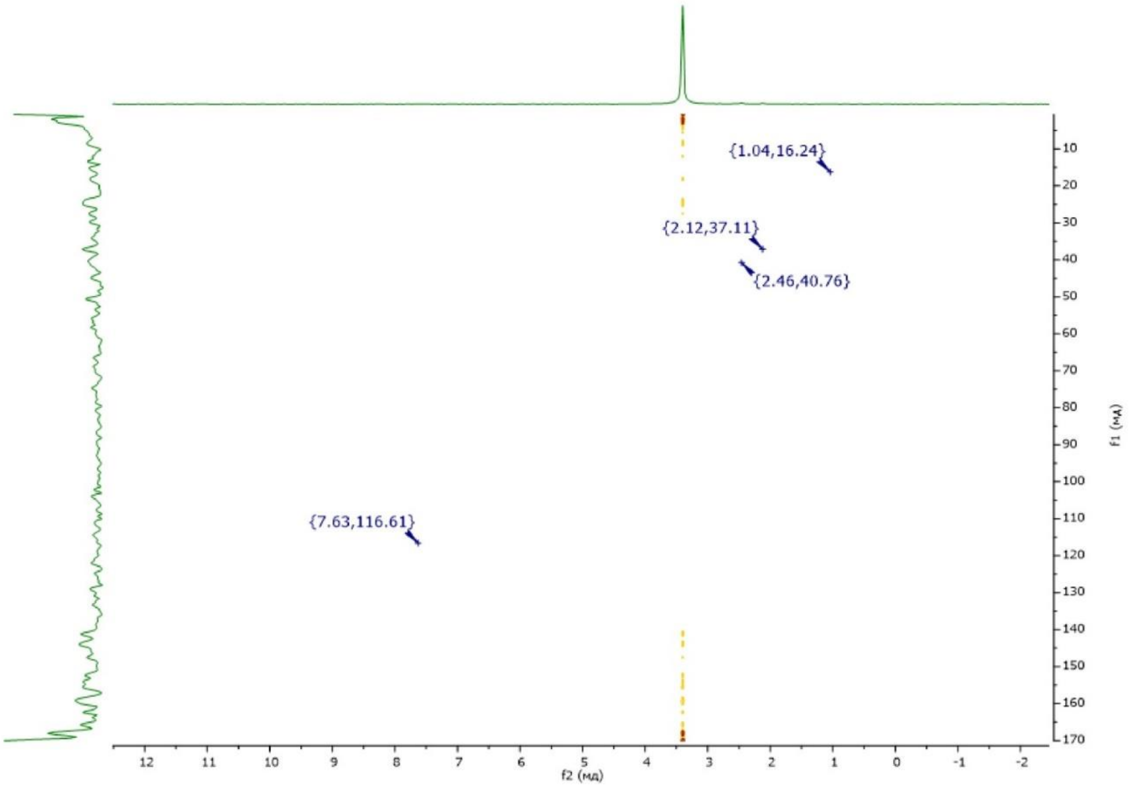

Figure S13. NMR spectrum HMQC of 1-(2-ethoxyethyl)-4-ethynylpiperidin-4-yl *m*-fluorobenzoate (LAS-286 base) in DMSO-d_6: H^{11}→C^{11}, $H^{3,5}$→$C^{3,5}$, $H^{2,6}$→$C^{2,6}$, H^{7}→C^{7}, H^{18}→C^{18}, H^{20}→C^{20}, H^{19}→C^{19}, H^{21}→C^{21}.

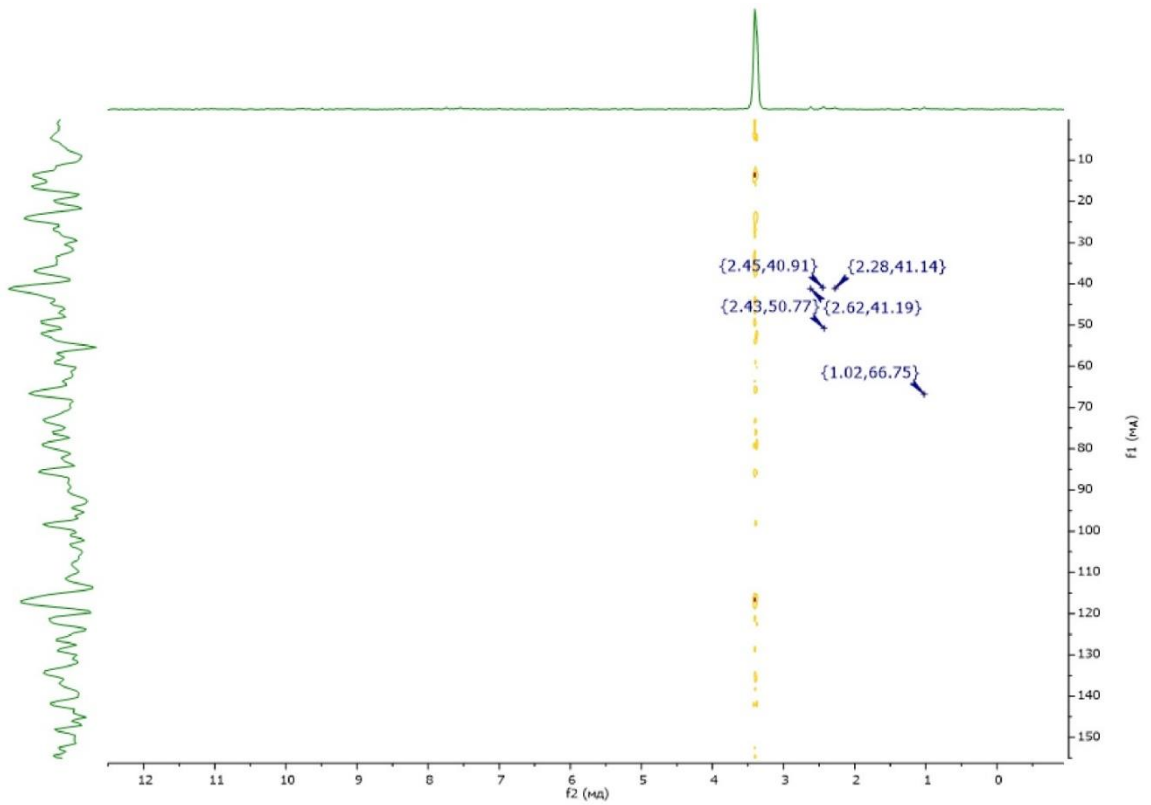

Figure S14. NMR spectrum HMBC of 1-(2-ethoxyethyl)-4-ethynylpiperidin-4-yl *m*-fluorobenzoate (LAS-286 base) in DMSO-d$_6$: $H^{11}\rightarrow C^{10}$; $H^{2,6}\rightarrow C^{7}$.

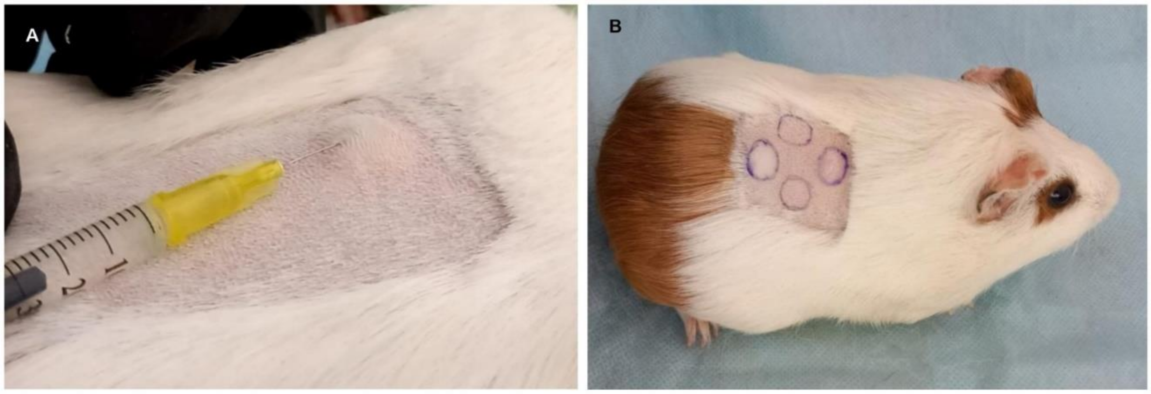

Figure S15. Intradermal administration (**A**) and the area of injection (**B**) of the test solutions (wheal method) on a shaved area of the back of a guinea pig according to the Bulbring and Wajda model.

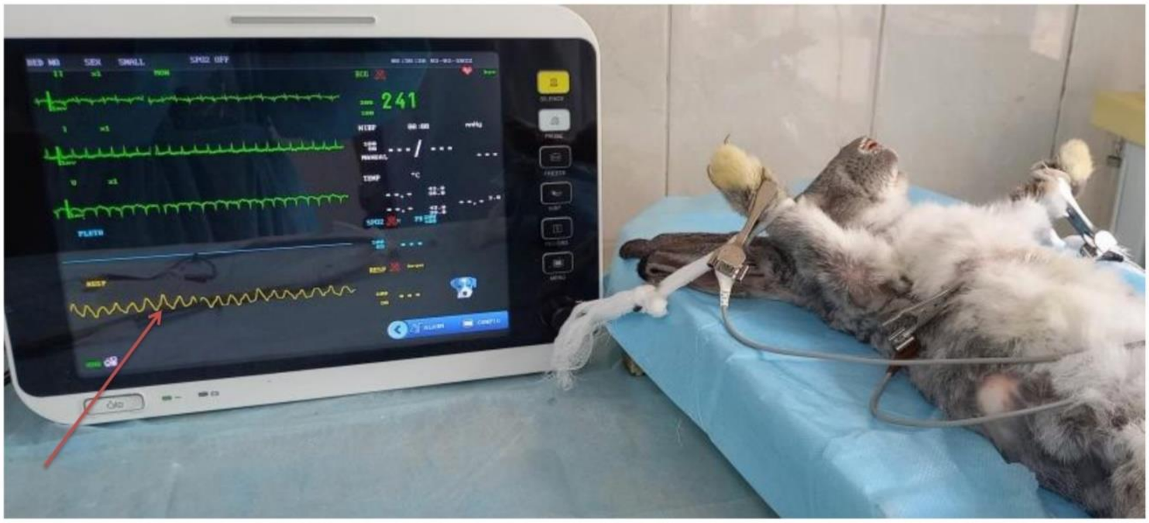

Figure S16. The process of recording changes in the rhythm and amplitude of breathing during the study of a local anesthetic activity during the infiltration anesthesia of the abdominal wall in rabbits.

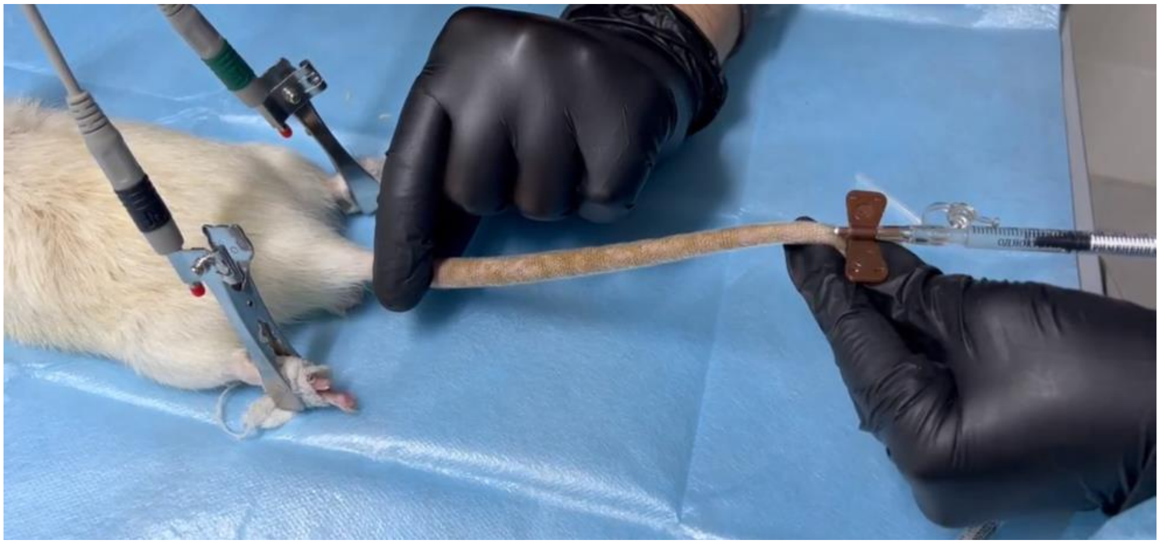

Figure S17. Administration of the aconitine solution into the lateral tail vein of a laboratory rat when studying antiarrhythmic activity.

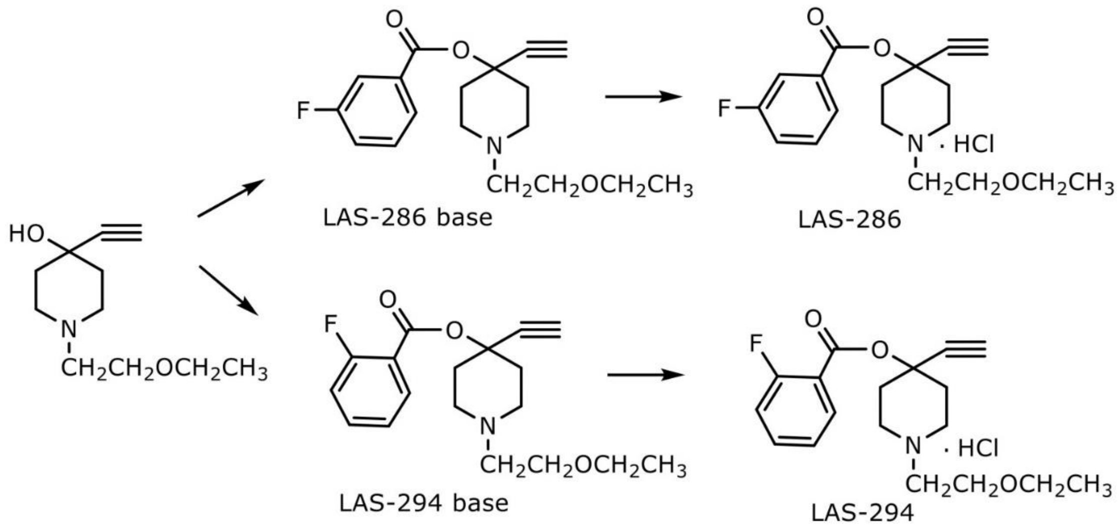

Figure S18. Process of obtaining the investigated compounds.

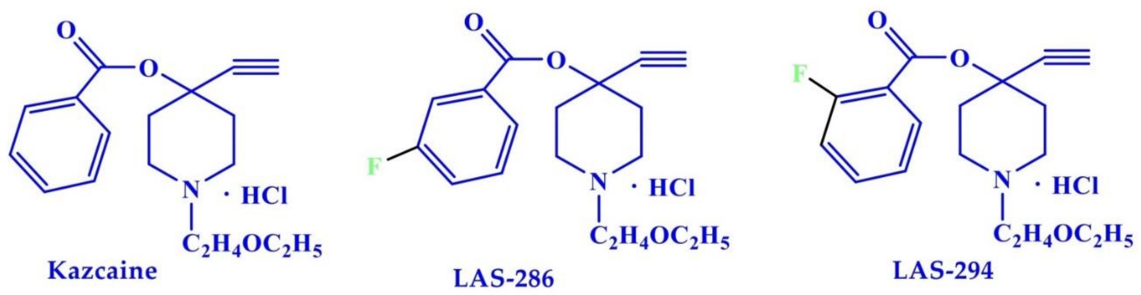

Figure S19. Chemical structure of the new piperidine derivatives.

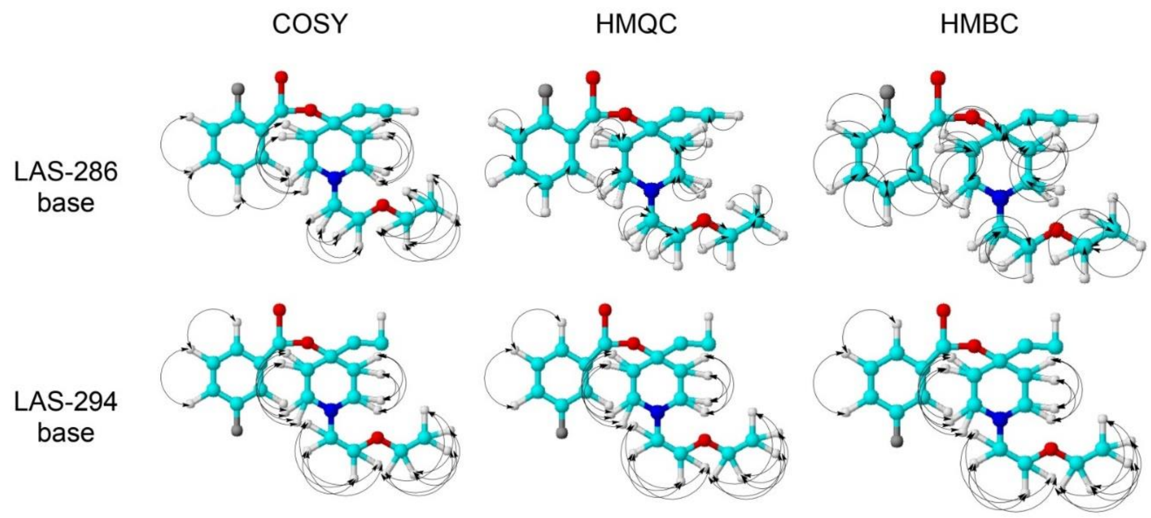

Figure S20. COSY (1H-1H), HMQC, and HMBC (1H-13C) NMR correlations in the molecules (LAS-286 base and LAS-294 base).

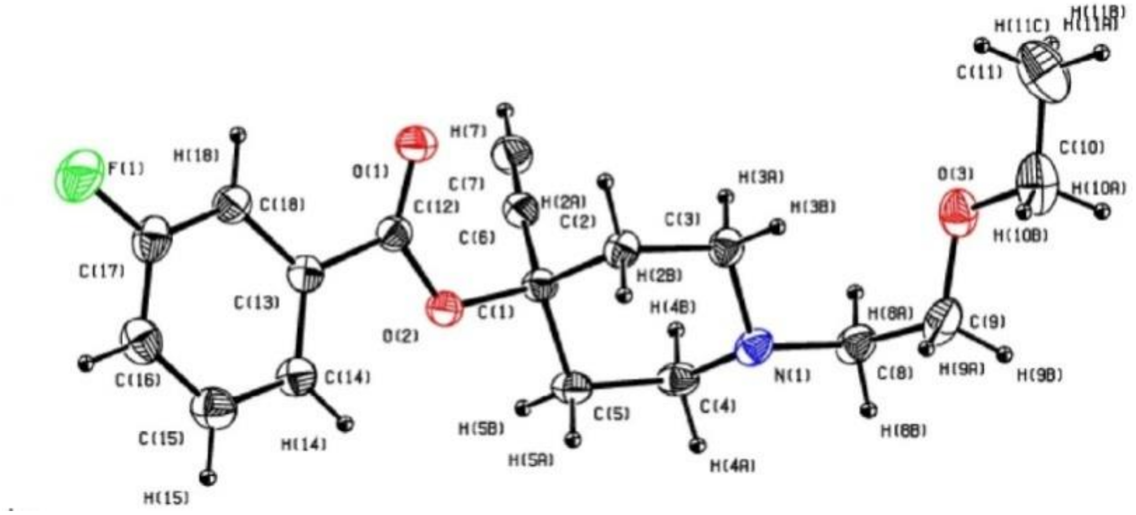

Figure S21. Molecular LAS-286 base. Thermal ellipsoids of non-hydrogen atoms are shown at the 50% atom location level.

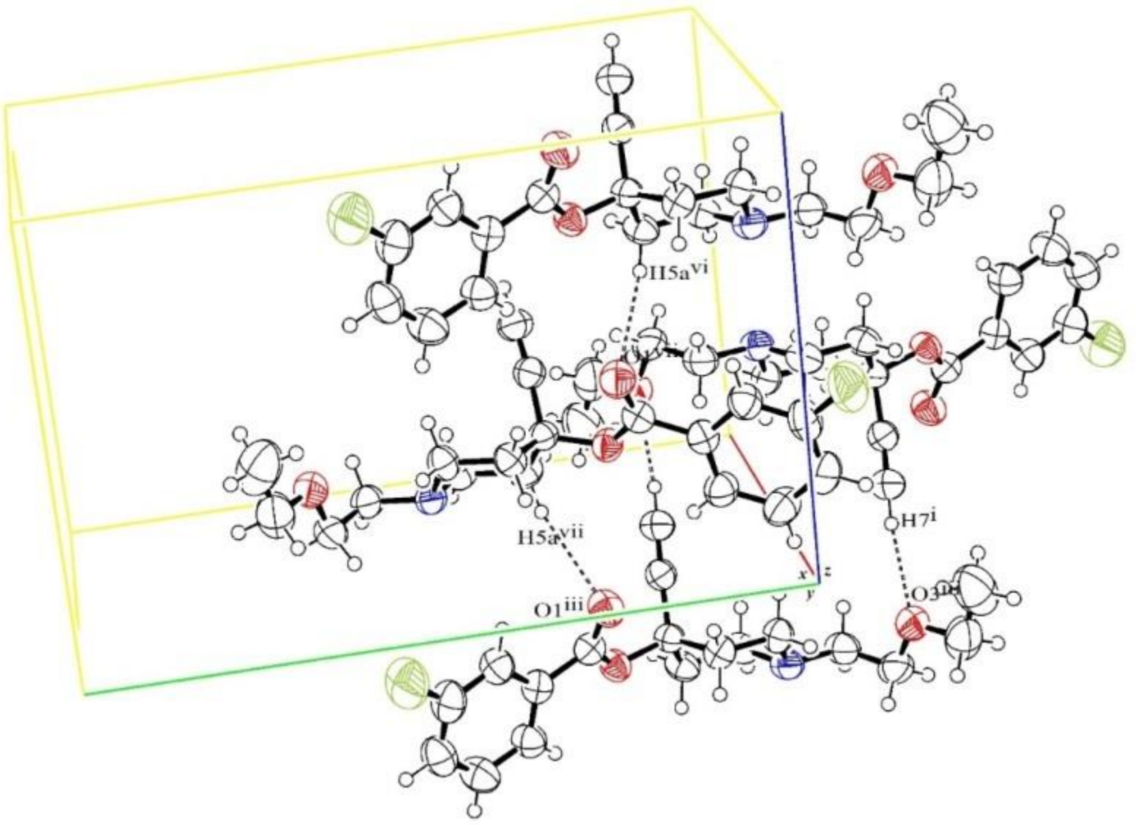

Figure S22. Crystalline structure of the LAS-286 base. The molecules in the LAS-286 base crystal are additionally connected to each other by the intermolecular hydrogen bonds (HBs) C(7)–H(7)…O3 (H…O 2.373 Å, C–H…O 173.67°) and C(5) –H(5A)…O (H…O 2.756 Å, C–H…O 160.90°).

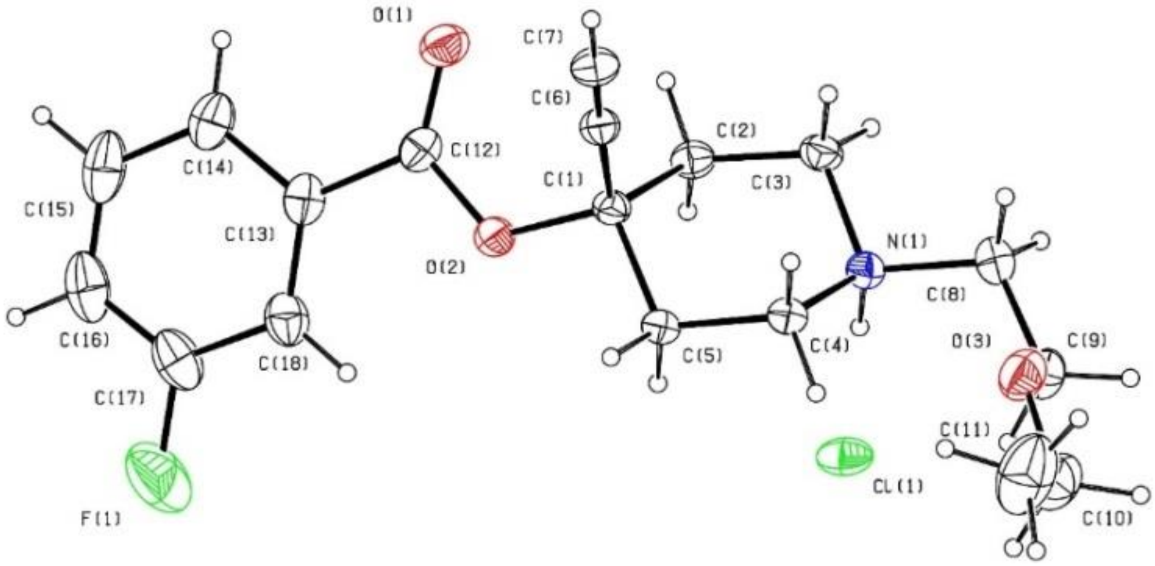

Figure S23. The molecular structure of LAS-286. Thermal ellipsoids of non-hydrogen atoms are shown at the 50% atomic location level.

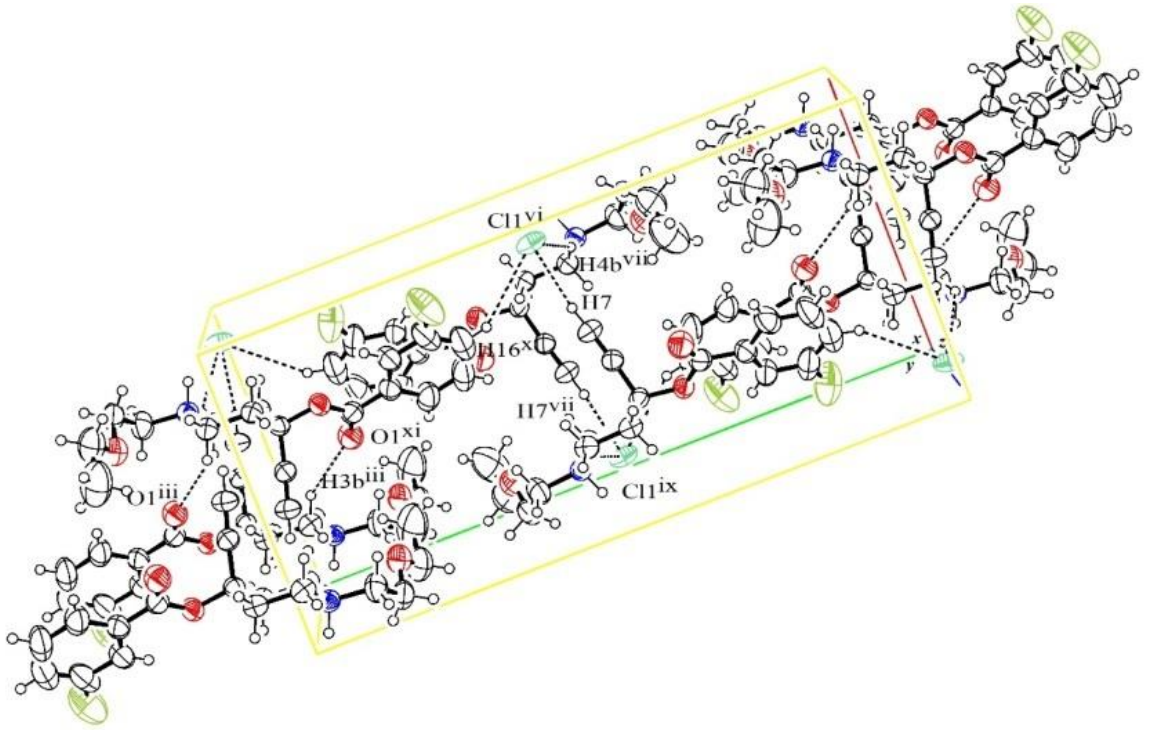

Figure S24. Crystalline structure of LAS-286. BC C(7)–H(7)…Cl(1) (H…Cl 2.5007 Å, C–H…Cl 159.01°) and N(1)–H(1)…Cl (H…Cl 2.0272 Å, N–H…Cl 174.36°).

Table S1. Basic crystallographic experimental data for LAS-286 base and LAS-286 structure.

Parameters	1-(2-ethoxyethyl)-4-ethynyl-4-(m-fluorobenzoyloxy) piperidine base (LAS-286 base)	1-(2-ethoxyethyl)-4-ethynyl-4-(m-fluorobenzoyloxy) piperidine hydrochloride (LAS-286)
Chemical formula	$C_{18}H_{22}FNO_3$	$C_{18}H_{23}ClFNO_3$
Formula weight	319.36	355.82
Temperature (K)	293(2)	
Crystal system	Monoclinic	Monoclinic
Space group	$P2_1/c$	$P2_1/c$
a (Å)	9.64700 (10)	10.05360 (10)
b (Å)	16.2937 (3)	21.9101 (3)
c (Å)	10.9138 (2)	8.72110 (10)
α (°)	90	90
β (°)	90.7301 (2)	95.9030 (10)
γ (°)	90	90
Volume $(Å)^3$	1714.71 (5)	1910.86 (4)
Z	4	4
$\rho_{calc}g/cm^3$	1.237	1.237
μ/mm^{-1}	0.750	1.979
F (000)	680.0	752.0
Crystal size (mm)	0.22×0.18×0.12	0.1×0.08×0.06
2Θ range for data collection/°	9.172 to 131.984	8.07 to 131.992
Index range	–8≤h≤11, –19≤k≤19, –12≤l≤12	–11≤h≤9, –25≤k≤25, –10≤l≤10
Reflections collected	16234	19029
Independent reflections	2980 [R_{int}=0.0338, R_{sigma}= 0.0241]	3299 [R_{int}=0.0218, R_{sigma}= 0.0134]
Data/restraints/parameters	2980/0/209	3299/0/218
Goodness-of-fit on F^2	1.058	1.085
Final R indexes [I>2σ(I)]	R_1=0.0643, wR_2=0.1953	$R_{1=}$0.0570, wR_2=0.1786
Final R indexes (all data)	R_1=0.0740, wR_2=0.2090	R_1=0.0614, wR_2=0.1837
Largest difference peak and hole $(e/(Å)^3)$	1.04/–0.33	1.01/–0.33
